# Diseases of the Urinary Organs

**Published:** 1842-10-01

**Authors:** 


### DISEASES OF THE URINARY ORGANS.

I. Traité Pratique sur les Maladies des Organes Génito-Urinaires. Par le Docteur *Civiale.* Tom. 3, pp. 1438, 8vo. Paris, 1837—42.

A Practical Treatise upon the Diseases of the Genito-urinary Organs. By Dr. *Civiale.* 3 vols. 8vo.

[Concluded from last Number.]

II. Lectures on the Diseases of the Urinary Organs. By Sir *Benjamin C. Brodie,* Bart. Serjeant Surgeon to the Queen. Third Edition.

### II.—Diseases of the Neck of the Bladder.

M. Civiale observes that the diseases of the neck and the body of the bladder have been confounded together; but, in fact, in by far the greater proportion of diseased states, it is the neck which is exclusively affected, and the body becomes so usually only secondarily. This part is liable to functional or vital lesions, as well as to those of organic character; and although, strictly speaking, perhaps, such distinction should not be made, yet, practically, it is required, and although the two classes of phenomena may become combined, and mutually influence each other, yet at other times they may exist independently.

### Chap. 1.—*Neuralgia of the Neck of the Bladder.*

The sensibility and contractility of the neck of the bladder are intimately connected, and the augmentation of the one produces that of the other. Until quite recently the most obscure ideas have prevailed upon these subjects, and even in 1834, it was objected, that so sensible a surface as the interior of the bladder would not allow of the injection of tepid water as a preliminary to lithotrity. The relative strength of the expulsive and resisting powers of the bladder, seated at its body and neck, vary in different individuals, and their derangement, in the various affections of the part, becomes the source of all the functional ills of the organ. In endeavouring to ascertain the extent of sensibility of the bladder, experimenters have confounded it with that of the urethra: but, after the vesical orifice of the urethra has been traversed, which occasions a sense of burning and a desire to urine, it will be found that no pain is excited by bringing the instrument in contact with the walls of the bladder, providing this movement be not rough or sudden. Indeed, as long as the mucous membrane continues healthy, even in cases of stone, pain is felt only by the patient when finishing the evacuation of the organ, the parietes of the bladder being then applied with force to the stone, driving it towards the orifice of the urethra. Many patients, who have suffered acutely from a small stone lodging at the neck of the bladder, have believed themselves cured when the catheter has driven the calculus into the body of the organ. The finger, passed into the bladder after cystotomy, causes pain only when it approaches the urethral orifice. The same fact is observed in cases of vesico-vaginal fistulæ and excessive dilatation of the urethra in women. But, although mere contact does not produce pain, pressure does. In its pathological condition the sensibility of the bladder becomes anormally acute, although its degrees and mode of manifestation vary infinitely, its effects being both general and local. But it is still at the neck, in the vicinity of the urethra, that the excess of sensibility is chiefly perceived.

Of all the sympathetic influences arising in these cases, that exerted on the cerebral organs is most manifest. Whether the lesion at the neck of the bladder be organic or not, almost every patient finds himself under the influence of a severe mental impression, giving rise to melancholy, and despair, while the encreased frequency of the desire to urine in proportion as the mind is turned to the subject, is well known. A habit of emptying the bladder when it contains but little urine, and a consequent state of irritability of the organ, is thus generated.

*Varieties of Neuralgia.*—(a.) *Simple Cases.*—In these the malady at present is entirely local, as manifested by pain and uneasiness about the pubes and perineum, frequent desire to urine, &c. The duration of the symptoms is variable, coming on usually in uncertain, but sometimes in periodical paroxysms. The intervals of ease become shorter, and the pain more severe and often extending to the hypogastrium, umbilicus, thighs, or even soles of the feet—its principal seat, however, being at the pubes or sacrum.

(b.) *Severe Cases.*—The simple case may have become only partially relieved by the means adopted, and an abiding state of suffering may continue, unaccounted for by the presence of any organic lesion. It is easy for the inexperienced to imagine a stricture exists here, and errors are frequently committed. Occasionally there is very little suffering in the genito-urinary organs themselves, but severe pain is felt in other parts of the body, especially at the umbilicus. Some of the cases are of an incurable obstinacy, and, although we may palliate them, we must not be persuaded to carry our endeavours at the cure too far. Many patients suffer principally or solely when subjected to the heat of bed at night, and many prevent their own cure by their impatience, and their having recourse to capricious modes of treatment.

(c.) *Complicated Cases.*—As the disease is usually subjected to empirical treatment, we seldom meet with it in its simplest state; but, when it becomes complicated with other affections, the neuralgia must be considered only as an accessory, exerting, however, great influence over the primary disease. Thus, *Stricture* is frequently so complicated; but, in the great majority of cases, the neuralgia disappears as the stricture is relieved. In these cases the difficulty of voiding urine is out of all proportion great, when compared with the extent of stricture, and great morbid sensibility sometimes remains after the latter is cured. Neuralgia is frequently combined with a rigid condition of the urethra; also with an induration of the glans penis, accompanied with slight stricture, and easily relieved by an incision of the orifice of the urethra. Affections of the prostate, fungi at the neck of the bladder, severe lesions of the walls of the bladder, are frequently accompanied with neuralgia, as a consequence, certainly, but yet demanding relief, before the primary malady can be attacked. Some of these cases seem incurable, while others cure themselves after all attempts at treating them are abandoned, and in all, palliation is to be obtained. *Calculous affections* are usually complicated with neuralgia; but it is surprising that with a foreign body irritating the neck of the bladder, the symptoms are not more constant, and are capable of even temporary relief. In another work the author has shewn how much the presence of neuralgia favours the production of gravel. The symptoms of mere neuralgia are so easily confounded with those of calculus, that Civiale has numbers of patients who apply to him under the erroneous belief of having a stone in the bladder. Even with the aid of careful exploration excellent practitioners have committed grave errors. The occurrence of neuralgia after lithotrity, sometimes gives rise to a fear of the reproduction of the disease. When the bladder is *hypertrophied* the neuralgic symptoms bear a still greater resemblance to those of stone, and great difficulty in deciding as to the absence of a calculus, exists, even when the injected bladder is examined. *Atrophy* of the bladder, occurring in feeble constitutions where there is a slow expulsion of urine, is frequently accompanied by neuralgia. The atony and neuralgia may exist independently of each other, but they are usually combined. We are often called to patients suffering from neuralgia, in whom, although there exists no organic lesion of the urinary apparatus, and the sufferings are comparatively slight, a *marked deterioration of the general health* is found to be present. A slight consecutive catarrh, accompanied with some atony of the bladder, is usually existing, the urethra is preternaturally sensible, and the urine high-coloured and fetid. These cases soon become very serious and require the most careful treatment. Diluent drinks, local baths, a prudent regimen, the wearing a suspensory, the administration of several glysters daily, and eventually the cautious introduction of the bougie and injection of the bladder, are the means to be adopted. Several months may be occupied in the treatment, which may be of a rather more stimulant character towards the end of the period. The least shock, by the injudicious use of instruments, conveyed to the system, will delay the cure, or even lead, at an advanced period of the disease, to dissolution.

*Causes of Neuralgia.*—The author does not consider the direct application of cold by injection or the douche, as one of these The various causes producing spasm of the urethra, *e. g.* contusion, undue pressure, irritation within the rectum, &c. may produce neuralgia of the neck of the bladder, but a leading cause of the malady is *resistance to the desire of passing the urine,* occurring especially during journeys, &c. when persons predisposed to spasm are very liable to retention, and this neuralgia is by no means uncommon in persons who have suffered from attacks in other parts of the body. The abuse of coition, and masturbation have frequently produced it, and no cases are more serious and obstinate. All practitioners are aware of the ill effects of fæcal accumulations, and, thus, mere glysters have succeeded in removing the affection. Constipation, and hæmorrhoids, which are often mistaken for venereal vegetations, fissures of the anus, ascarides, or even a slight phlegmon of the extremity of the rectum, may exasperate the sensibility of the neck of the bladder.

*Influence of Age and Sex.*—Although it is chiefly among adults that these affections are found, they exist in the young and oftener still in the aged. In *children* the symptoms are so varied as seldom to be specially characterised, and they oftener cease of themselves rather than in consequence of treatment. In the *aged* they are usually confounded with the organic lesions found at this period of life, of which, indeed, they are usually but consecutive or complicatory. Although neuralgia of the neck of the bladder is far from uncommon in women, yet, as their modesty prevents an early application for treatment, we usually find some serious complication existing when called to them. It is often mistaken for an affection of the neck of the womb. The author has met with many cases of aged women, worn out by fatigue, labour, or misery, who suffered excessive pain during the passage of the urine, which was accompanied with catarrh of the bladder, and general deterioration of health. Palliation, by procuring a freer expulsion of urine, is all these cases will admit of.

*Treatment of Neuralgia.*—The distinction of two classes of cases, the one dependent upon a mere exalted vitality of the parts, and the other arising from the structural changes, is to be borne in mind; for, although the neuralgia may become relieved by similar means in both instances, this is to be regarded as permanent in the one, but only as temporary in the other, as long as the original cause endures. In the *simple* cases, every or every other day, according to the degree of irritability of the patient, a bougie should be introduced into the urethra, for from five to ten minutes, removing it at once if it cause uneasiness, for the success of the practice depends upon the slowness and caution with which it is conducted. In others, besides the most simple cases, *injection* of the bladder will be required after the bougie has been employed for some time, commencing first with tepid and gradually arriving at cold water. Further observations upon this matter will be made when speaking of the atony of the bladder, the complication which renders injection necessary in neuralgia. *Douches,* applied to the various external parts of the pelvis are too much neglected. They may be formed at first by warm water, (mineral water is often very useful,) and afterwards by cold, and as the unpleasant feeling from their use arises from the water splashing on the neighbouring parts, these may be covered. They should be continued from ten to twenty-five minutes. Free action of the bowels, by glysters and mild laxatives, emollient drinks, and frequently the production of a pustular eruption are often desirable means. But all attempts to act through the bowels and skin have little effect, until we have modified the sensibility of the neck of the bladder by the bougie. The cure is sometimes very rapid, for passing a bougie two or three times has relieved many patients. The simplicity of this treatment induced the author to instruct his patients how to use the instrument; but he repented having done so, as they returned to him worse than they were, having been too rough in their manipulations. All attempts at hurrying on the case, or the substitution of the catheter, will be found fruitless. The patient's inattention to regimen, and other means of cure, together with his depression of mind, and unwillingness to undergo the necessary examination, often dangerously delay the treatment and render him liable to exacerbation or relapse. Medical men especially make bad patients, by reason of the mental torment they inflict upon themselves, and their tampering with the remedial means proposed. The circumstance of the difficulty of excreting the urine being so much under the influence of the mind encreases their sufferings. Owing to the dislike which patients feel in applying concerning the maladies of the genito-urinary apparatus, the affection has often made serious inroads upon the health, under which circumstances, any slight shock to the system, as from a journey, change of diet, &c. will be sufficient to bring on an attack out of all proportion severe to the patient's apparent condition prior to its occurrence, and which must teach us to be careful in our prognosis, especially as the patient and friends will be too apt to attribute to the means we employ the effects which they observe. *Depletion*, when a phlegmasia has been supposed to be present, has sometimes been carried to an enormous extent, and although the author considers applying a few leeches to the loins and perineum may be sometimes useful, he condemns the application of 200 to 1000, which he has seen had recourse to. *Opiates* should only be used as a means of relieving pain or suffering, to favor the application of direct means of cure; but when injudiciously employed the disease may become masked and difficult of recognition in its various complications, which thus have time to develope themselves. Gout, rheumatism, and a variety of diseases, especially syphilis, have been in turn charged with the production of neuralgia, and then various remedies vaunted for its cure. The history of the patient's life is ransacked, and if ever he has during it suffered from syphilis, that is considered an explanation, which the prepossessions of the patient himself tend to render satisfactory. The consideration of the varying quantity and quality of the urine has also given rise to much empirical treatment, especially in England. The obstinacy of some cases has led to the employment of caustic, which, if carefully conducted, is unattended with danger. The author has frequently used it, but not until all other means have failed, sometimes with good results, but usually without any marked advantages. He applies it in the slightest possible manner, for when it is too boldly employed numerous and serious evils may result. The diet must be carefully attended to, the urine maintained in a fluid state by diluent drinks, atmospheric vicissitudes and long journies forbid. While, as a general rule, abstinence from coition must be insisted on, a too long continence, on the other hand, has been found hurtful.—*Relapse*. Although in many cases it suffices to cure the disease without taking any subsequent precautions, save the avoidance of the primitive causes, yet, in others, we find the ailments reproduced without obvious cause, although usually in a diminished degree. We should therefore not discontinue our remedies at once, nor until some time after the apparent cure has occurred; and the tendency to relapse is so strong that it will sometimes occur in spite of every precaution.

Chap. 2.—*Affections of the Vesiculæ Seminales and Ejaculatory Ducts.*

The obscurity of this subject, by reason of the difficulty of its diagnosis from various maladies of the vicinity, is well known. Neither the local symptoms or general disorders present signs sufficiently distinctive.

1. *Changes found after Death.*—The author conducts his post-mortem examination of the genito-urinary organs as follows. Insisions on each side of the body are carried from the external margins of the abdominal ring, so as to meet each other at the posterior portion of the anus, and the pubes and ischium are to be divided in the same direction. The kidneys must be detached without dividing the ureters, and having made an incision through the intestine at the sacrum, the contents of the pelvis may easily be removed. Having placed the parts upon a table, the portions of the pubes and ischium which had been sawn through, are to be removed so as to expose the anterior part of the bladder, the upper surface of the prostate, and the deep portion of the urethra. A catheter is to be introduced, and having punctured the bladder at its fundus, an incision is to be extended through its body, neck, and the membranous portion of the urethra to the root of the corp. cavernos. The lateral and lower parts of the urethra must be left undivided, for it is here the chief alterations will be found, care being always taken at once to examine the orifices of the seminal canals. In many of the examinations which the author has made of persons who have died after retention, or other affection of the urinary organs, he has found great dilatation of these orifices, and that large quantities of pus issued upon pressure of the vesiculæ seminales. The changes in the vesiculæ are those usually found as the result of inflammation, leading to disorganization; it terminates sometimes by suppuration, but usually by induration or even ossification.

2. *Diagnosis.*—This is full of obscurity, some coincident affection often masking by its gravity the primary one. A principal sign of a phlegmasia of these organs is a precipitate ejaculation of the semen either during coitus or dreaming, the fluid being expelled before the erection is complete, or even while the penis is flaccid. The same, however, may be the result of vicious habits, and some diseases of the testis or neck of the bladder; but when it occurs in an eminently nervous subject, and is reproduced very regularly, it usually arises from an affection of the vesiculæ. Another symptom, said to be diagnostic, is the sense of uneasiness, a painful yet vague description of fatigue, which accompanies or follows each emission, and which may continue hours or even days. This is also found in some affections of the prostate, and after the abuse of venereal pleasures, but not so distinctly as in the present case. Great changes also occur both in the quantity and composition of the fluids emitted. Some consider the superabundant discharge to be semen, and others as the production of the irritation of the prostatic portion of the urethra. In many of these patients, indeed, it is to be presumed, from their age, the prostate is affected, and small emissions are provoked by the slightest exertion, going to stool, &c.; but yet this must be considered as distinct from the constant running found in prostatic disease. When, however, the urethro-prostatic discharge is mixed with semen the case puts on a far graver aspect than when this is not the case, as the effect upon the general health is infinitely greater. Indeed, so serious do the secondary disorders then become, that the attention of the practitioner is diverted from the source of the mischief. Patients, in whom there is but a slight discharge, in whom the spermatic passages are but little affected, pollution occurring rarely, are often menaced by the most alarming deterioration of the general health, their minds are utterly dejected, coition becomes impossible, and the semen is discharged without erection or sensation. These patients will be found, upon careful examination, to have been long liable to these small discharges, which, perhaps mixing with the urine, have not been attended to by them. In these affections we see the marked sympathy between the genital and cerebral organs: melancholy, often leading to suicide, takes possession of the patient, and partial tremors of the eyelids or hands, eventually converted into complete convulsions, are present, while, after death, some lesion of the encephalon is not unfrequently discovered.

3. *Causes.*—By reason of their position and relation to other organs these parts are liable to be influenced in a variety of manners, each of which impresses some special mark upon the affection, and exacts a variety of treatment. But authors have enumerated many circumstances which are of trifling or imaginary operation, and have overlooked others of real importance. Much importance has been attributed to *gonorrhœa* as producing the affection by a direct transmission of the inflammation, but upon no good grounds. Indirectly it may have some influence by the production of stricture, irritation of the neek of the bladder, &c. The ejaculatory ducts, opening just behind the narrowed portion of the urethra in *stricture,* may participate in the lesion, and this our author believes to be one of the most frequent causes of the affection. He considers various empirical means employed to procure the cessation of discharges of the urethra, as astringent injections, caustic, the rough use of instruments, &c. powerfully tend to the same end. *Irritation and inflammation of the prepuce and glans* also re-act upon the seminal vessels, and produce an altered or superabundant discharge, which becomes relieved as the phlegmonous state of the prepuce subsides. When the glans is reddened and indurated, the consequent lesion of the neck of the bladder is sometimes attended with an incurable discharge. The excitement to erection, which the irritation of the prepuce produces, causes a more frequent recurrence to coition, and at first an increased to be followed by a diminished secretion from the testis. The influence of a diseased state of the *prostate* cannot be doubted. Disease and irritation of the rectum it is well known produce irritation of all parts near the neck of the bladder. Many children are at first incited to masturbation by the irritation produced by ascarides, &c, ; obstinate diarrhœa and irritating purgatives may have some influence also. Masturbation and excess of coition, especially if the patient have become addicted to these before the sexual system be fully developed, are frequent causes. Although these affections are frequently found to exist in members of the same family, the author does not consider their hereditary nature as at all established.

4. *Medical Treatment.*—Usually the most dominant symptom is attacked without any reference to the nature of the disease. The author considers its treatment can be best illustrated by dividing it into three different degrees. In the first, where there is too rapid emission followed by prostration, defective erection, and nocturnal pollution, the urethra will be often found preternaturally sensitive, and by relieving this cautiously by means of the bougie a cure of all the symptoms is sometimes obtained. But the neuralgia here is much more obstinate than when it exists in a more simple state. The great object, and the great difficulty is to commence the treatment early, as the practitioner is not called in until the case has made too much progress. One reason that leads to delay is, that as within certain limits, exercise increases the activity of function of an organ, many young men, finding their virile powers only increased by coition, do not perceive, until too late, when they have arrived at excess, when the power becomes diminished, and the surgeon now first consulted, often finds a phlegmasia of the vesiculæ firmly established. Again, these patients, finding even after the power of erection has ceased, that frequent emissions occur from the irritation excited in the testis by the phlegmasia, attribute their condition to prolonged continence. When the affection arises from onanism its cure is yet more difficult. In the *second* degree, when the power of coition is entirely lost, and emission occurs without sensation, and is followed by excessive prostration, a mucous discharge flowing from the urethra, especially during stool, the treatment is more difficult. In these cases the removal of all causes of irritation from the rectum is highly desirable, and the production of easy evacuations by glysters advantageous, and all drastics are to be avoided. Ascarides not only infest the rectum, but may exist in the external organs of generation, especially in young girls, when they are often overlooked. The state of the urethra must be particularly examined, and its neuralgia, when present, carefully treated. The application of *caustic* to its deeper portions produces sometimes a cessation of symptoms which have resisted all treatment. But the urethra must have been well prepared, and its anormal sensibility diminished by the use of the bougie, and the application must be made in the slightest manner, when the re-action which always ensues will soon pass away. A month should elapse before the second application, and a third is rarely advisable. It is not to be used at all until all milder means have failed, some of which have been too much neglected, as *e. g.* frequent glysters of cold water, and the persevering use of the douche, &c. He does not think much of counter-irritation. The full *diet* the patient's sense of debility often induces him to take only adds to his distress; it should be of the mildest kind. The lying in hot beds, frequenting assemblies, neglect of rest, commerce with women, riding, &c. are to be prohibited. Pedestrian exercise is useful, and the engaging the patient in a regular train of study, which is rarely practicable, might be of advantage. *Tonics*, and especially iron, are useful in one class of cases, but in another in which the debility is merely consequent upon a phlegmasia not yet relieved, they are very hurtful. The preparations of *lead*, also sometimes useful, have often done harm from indiscriminate use. When all means have been fruitlessly tried, a *third* degree of the affection may be said to exist, in which desire itself becomes extinguished, and the patient is weighed down with the conviction of hopeless impotence; the mere state of inflammation is no longer to be combated, but a work of local destruction, accompanied by a shattered state of the general health, under which the patient may rapidly sink into one form of what has been called consumption.

5. *Hygienic Treatment.*—(1.) *In excessive Action of the Organs.* This, producing a difficulty of ejaculation, and, by re-action on the testes the secretion of an irritating semen, is rarely original, but is usually provoked by masturbation or venereal excess, and then becomes amenable to treatment, providing the original error be detected, which it is often with difficulty, owing to the way in which its existence is pertinaciously denied. (2.) *In defective Action.* Excessive *continence* may give rise to evils analogous to those proceeding from an opposite cause. It may be practised after debauchery, and then the system suffers by reason of the suddenness of the change : or it may have always existed, as with Newton and Pascal, when evil may result in two modes. If a man, who has always been continent, suffers from vivid desires and frequent pollutions, the former will after a while become diminished in proportion as the latter are increased, and what was at first but a natural relief degenerates into a vicious habit, capable of leading to all the ill consequences of masturbation, and if the patient acquire the habit of emitting without pleasurable feeling he will not be enabled to perform the functions of a husband. But a man is in no better condition if his desires are feeble, and his night-emissions rare, for this shews an inactivity of the genital organs, which want of exercise only serves to encrease. Such a man may boast of his continence, but this arises rather from the absence of desire than from his vanquishing it, and it may be questioned whether organs continuing so long in a condition of inactivity, will assume a requisite degree of vigour when required. As a practitioner gains his patient's confidence he frequently finds the abstinence from women arises from the presence of more or less impotence or the want of confidence in his virile powers, rather than from religious motives. A man in health feels invigorated after coition, and more fitted for mental and physical exertion, and whenever the act is followed by a feeling of melancholy or debility, inaptitude for exertion, and an irresistible desire for sleep, coition has usually exceeded its limits, and some one of the genital organs is suffering in consequence.—(3). *Determination of the Aptitude or Inaptitude for Marriage.*—This in these cases is often a delicate question: for, while in some cases, marriage, by removing the temptation to solitary enjoyment, and regulating the natural functions of the genital organs, becomes the best means of cure; in others, the excitement inseparable from the union of the sexes becomes hurtful, and may alone reproduce all the evils that treatment has relieved. With the genital organs, as with others, exercise fortifies, inaction enervates, excess diminishes and destroys their power. Thus, when they have become enfeebled by abuse, repose is their first essential, after which a gradual and moderate return to their natural excitement, proportionate to their renewed energy, is the rational course to recommend. In considering this subject it must be remembered, that virility and the development of the external organs are not necessarily proportionate. There are several accidental circumstances which are supposed to exert an influence upon the powers of the genital organs. Thus, a *dilatation of the spermatic veins* has been supposed to lead to sexual debility, and certainly the testis on that side may remain undeveloped, or become atrophied. *Malformation of the penis* also, by not only impeding congress, but by proving a source of irritation, re-acting on the testes and vesiculæ may produce the same debility. But such debility is often only coincident, and many men possess full energy in spite of the malformation. The genital organs are sometimes found to continue in a *rudimentary state,* under the operation of some unknown cause, and the individual usually exhibits characters resembling those of the opposite sex. A *disproportion* of size between the *glans* and *the body of the penis* demands notice. The latter may be long, flaccid, and pendant, while the former is dense and voluminous. This is rarely primitive, but usually arises from the abuse of coition or onanism, and it is very rare for these cases to be restored to their normal condition, especially if the testes have become soft and the scrotum relaxed, the erections continuing feeble and incomplete. The pressure of a badly-applied *truss* upon the cord has produced a diminution of the size of the testis. Several anormal conditions of the *testicle* are observed. It may be knotted, tuberculous, soft and flabby, or entirely or partially indurated; the epididymis may be disproportionately large; the cord is sometimes lank and soft, at others hard and knotty. Very frequently these individuals are subjected to continued discharges, or involuntary emissions of a fluid very unlike semen; and they are usually little apt for the generative functions. The existence of *desires* on the part of the person has erroneously been supposed to be an index of his power of accomplishment. These are much more dependent upon the condition of the brain, than upon a state of repletion of the seminal reservoirs. These desires are often found to exist strongest as the procreative power diminishes. The mere existence of *erections* proves little; they may arise in some cases after the use of carriage exercise, hot baths, indulgence in hot, soft beds, &c. They may occur in morbid conditions of the bladder, as *e. g.* where the urine has been too long retained in neuralgia, usually subsiding when the bladder has been evacuated, but sometimes requiring cold to subdue them and permit the urine to pass. They are most obstinate towards morning. They are found too in old men and children. Morbid erection may also arise from the excitement produced by excessive coition; and when men above a certain age marry young women they frequently become liable to them from the excessive demands made upon their powers. These cases are easily distinguished from the natural erection as *ejaculation does not terminate them,* but they often become more prolonged in proportion as the act is repeated, and are frequently accompanied by a burning sensation. Patients, believing that renewed coition is the only remedy for the state of erection, are astonished when entire repose is prescribed. Reading lewd books, and frequenting the society of loose women, lead also to the production of erections which express no real want.

6. *Affections of the Genital Organs in Children.*—Even in very young children these are liable to augmented susceptibility and diseased conditions, the detection of which is rendered the more difficult, by reason of the want of development of the parts. Worms in the intestinal canal, and especially early masturbation are principal causes. This habit, at an early age, is the more to be dreaded because it does not result from the existence of a real want, but from a fatal precocity of the nervous system, and is hence chiefly manifested in delicate, excitable, nervous children, the development of whose intellect is dangerously premature. A considerable discharge often occurs in boys and in girls, and the habit of producing it may become incorrigible, but in some cases it is very slight and curable.

The excess of sensibility of the genitals which precedes the discharge is usually overlooked, and this latter is treated empirically, and if recognized at last, when alarming sympathetic maladies are engendered, it is usually too late. Cleanliness of the penis is too much neglected in children, and the accumulation of sebaceous matter under the prepuce, by the irritation it produces, causes the child, as one suffering from stone, to pull the penis, and thus often leads to masturbation. When young persons arrive at puberty such irritation often produces sympathetic prostatic discharge, to relieve which effectually circumcision is sometimes required.

The treatment of these cases is more difficult than in the adult, as it is more tedious. The bougie and caustic are less efficacious here, and sometimes an instrument has to be retained for some time. The cold douches are useful, and the condition of the rectum requires especial attention.

7. *Affections of the Genital Apparatus in Women.*—Medical men are not often consulted directly upon this subject by women; but such cases are of frequent occurrence among young girls, young wives separated from their husbands, and widows. As the practitioner can only usually suspect evil practices, being debarred direct questions upon the subject, he has, too often, only to treat symptoms although the general health continues to fall away. Excessive coition will give rise to the same effect as onanism. Many women who practise these excesses are to be pitied rather than blamed, for in them an exaggerated and frequently a preternatural development of the genital organs is found, causing a continuance of the sexual desire in some also long after the period at which it is usually extinct. Still there are several examples in which the functions of the generative organs are unusually active unaccompanied by any proportionate development. With some women the same ill effects proceed from *continence,* and are at once removed by marriage.

### CHAP. 3.—*Urethro-Prostatic Discharges.*

The mucous membrane of the urethra may become the seat at various points of a special phlegmasia, hardly frequently to be distinguished by its symptoms from neuralgia, but giving rise to a peculiar discharge. This, however, varies in quantity, colour, odour, and consistency—the linen with which it comes in contact seeming to have been gummed, the spots being of a reddish or bluish colour. Patients suffering from this discharge, when abundant, often observe a redness and constant humidity of the orifice of the urethra, and when not caused by external irritation, it is an index of chronic phlegmasia of the deep-seated portion of the urethra and the vesiculæ seminales. The discharge may come on after unusual mental or corporeal exertion, excess of coition, the means used to arrest a gonorrhœa, or the too liberal use of fermented liquors, especially beer. Obstinate cutaneous eruption may co-exist, and disappear only with the discharge. Small calculi lodged in the deep-seated portion of the urethra, or between the prostate and rectum, by the irritation they cause, may keep up the discharge. Frequently, after death, great dilatation of the orifices of all the ducts opening into the urethra, and sometimes the development of little sacs in the canal, are found. Affections of the rectum play an important part, and thus hæmorrhoids in old men, often produce much urethral discharge, as also do affections of the womb. Too often it arises from organic changes in the neck of the bladder and prostate. If the discharge be not excessive it is not dangerous; some old men have, like women with leucorrhœa, suffered little else than an impediment to cleanliness for years. But, in many cases, the powers of the digestive organs give way, and excessive pain attends the expulsion of the urine. In treating the case we should search for the cause, and not treat the discharge in an exclusive manner.

CHAP. 4.—*Affections of the Veru-Montanum.*

The author cites several examples, derived from his own and others' experience, of organic change in this part. The symptoms are often severe but wanting in special character. The use of astringent injections or bougies, or the irritation arising from enlarged prostate, or a calculus have been produced as causes. Its exploration is difficult to the inexperienced, and its treatment, when advanced, should be that of tumor of the neck of the bladder.

CHAP. 5.—*Bridles at the Internal Urethral Orifice.*

A contraction of the vesical orifice of the urethra may exist in a membranous or cord-like form, raising the mucous membrane as a valve at its lower margin. It is usually placed at a short distance from the verumontanum, with which it becomes confounded. The mucous membrane is of a violet colour, as if from a phlegmasia. If viewed from the bladder, prior to dividing the prostatic portion of the urethra, the valvular nature of the obstruction, sometimes nearly concealing the orifice, may be well seen. The symptoms are obscure, but a careful exploration, made at an early stage of the affection, before the prostate and other parts at the neck of the bladder become implicated, will reveal much. True stricture here is rare. When the diagnosis is clear, the bougie, sound, or caustic, or carefully directed incision may be employed, and for this last the author proposes an instrument in lieu of the one invented by Stafford.

CHAP. 6.—*Varices at the Neck of the Bladder.*

Although hæmaturia, retention, &c. have been attributed by some to the existence, at the neck of the bladder, of a similar condition of the veins, to that which prevails in those of the rectum in hæmorrhoids, the author agrees with Shaw, that no such a state of these vessels prevails. A great distention of the capillary vessels of the bladder occurs from many causes of irritation.

III.—DISEASES OF THE PROSTATE.

CHAP. 1.—*Enlarged Prostate.*

The author gives a long description of the various modes in which this may occur, which we have not space to follow. Suffice it to say, that it is rare in old age to find the gland in an exactly normal condition. It may be swollen generally, and then may reach extraordinary dimensions, or the central portion, or lateral lobes, may become separately affected in every variety of extent.

*Effects* (a) *upon the Urethra.*—The mere tumefaction of the lateral lobes does not necessarily change the direction of the urethra, but the deprivation of the suppleness and elasticity, which belong to the urethral walls, sufficiently explain the difficulty the patient experiences in urining. When, however, these lobes are unequally swollen, or, when one only is affected, the canal becomes diverted from its normal direction, and the projection of the enlarged lobes into the bladder produces a distortion of the orifice. It is by the enlargement of the central portion that the greatest change in the urethra is effected, the deviation of the canal upwards varying in form, extent, and regularity. The encrease of the gland also, by thrusting the neck of the bladder backward, and the membranous portion of the urethra forwards, encreases the length of the prostatic portion of the canal. (b.) *Upon the Bladder.*—These occur in various modes, first, as to its capacity, affected not only by the volume of the gland projecting within the organ, which in some cases is enormous, but also by the contraction of the bladder its irritating presence produces. The form of the bladder is also much changed, a notable excavation, as it were, existing at the bas-fond; the various changes in this respect being only appreciable when the use of exploratory instruments is attempted. The functions are much disturbed. The expulsion of urine, even when the power is preserved, becomes very painful, and the organ itself very irritable. The spasmodic contractions are often dreadfully severe, and the contact of an instrument with the walls of the bladder gives rise to intense suffering, and sanguinolent urine, or even blood itself may follow the efforts made. After death, hypertrophy, accompanied with more or less phlegmasia of the mucous membrane, is found. In robust patients we find the state of hypertrophy and encreased capacity of the bladder conjoined: but, in the feeble and exhausted, distention of the organ is caused by complete atony. (c.) *On the Kidneys.*—Inflammation and consequent abscess is frequently induced indirectly, by reason of the difficulty occurring in the expulsion of the urine. (d.) *On the Genitals.*—Most persons, suffering from chronic affection of the prostate, find procreation difficult. Usually there is a gradual diminution of desire, coition is performed with less and less frequency, attended with less pleasure, and is followed by distressing prostration. Sometimes the testes are soft, atrophied, and pendant, but at others, uniform and consistent; but in all cases usually very sensitive. The catheter, which is required to empty the bladder, keeps up the irritation in these organs, and the case often becomes incurable. Many castrations have been improperly performed under the idea of special disease of the testes existing. In many cases this organ may regain its usual size, and all tenderness cease, but there still remains deep-seated pelvic pain and general uneasiness, due, probably, to an affection of the vesiculæ seminales.

*Diagnosis.*—The *symptoms* assist us but little. The affection may have existed a considerable time without being denoted by any very marked, so that, when called to the patient with retention of urine, we find the disease has made considerable but unheeded progress. Sometimes, however, they are markedly severe, but not always easily referrible to their proper cause. Every variety of difficulty of urining may exist, but it is rarely found to be proportionate to the gravity of the case, and indeed frequently surprisingly the reverse—so that we must not attempt to explain either the existence of retention or incontinence by mere post-mortem inspection. The imperfect character of the jet of urine throws no light on the case, for while this may exist in any stricture, it may also be due to a defective expulsive power of an atonic bladder. The urethral discharges do not present any distinctive appearance. The ribbon-like form of the fæces can only occur in those rarer cases wherein the tumor takes the direction of the rectum, but when the gland becomes voluminous, and produces irritation of the gut, the patient suffers from pain and tenesmus at stool. As the symptoms afford so little information, *exploration* must be had recourse to. The rectum is to be examined by the finger, by which means we may learn the condition of the lateral lobes, but when the body of the gland is swollen, and carried toward the rectum, we must take care and not mistake it for some disease of the gut. A sound should always be placed in the bladder before examining the rectum, and when the bladder is distended the gland is forced lower down than it would otherwise be. The author gives full directions as to the information to be derived from the manner in which a catheter will pass into the bladder under the various forms of prostatic enlargement. But the difficulty of distinguishing, whether the change in the direction of the instrument arises from diseased prostate, or other causes, is often very great, especially when one sees the case after other practitioners, and false passages may be present and organized.

*Causes.*—The irritation caused by the contact of a calculus, the difficulty of urining produced in stricture, violence done in passing instruments, and the incautious use of injections may be among these. The attributing lesions of the prostate to venereal excesses has been done perhaps too hastily, for there are an immense number of cases to which no such cause can be assigned. Men of letters, who are but little inclined to sexual commerce, are very liable to prostatic affections, while these also occur in the positively inert. In some feeble young men, in whom the venereal functions have never become manifested, the prostate has continued rudimentary, and in some aged but continent persons it has been found atrophied, as if from chronic phlegmasia. The influence of *gonorrhœa* in producing affection of the prostate is very doubtful. The influence of *age*, from whatever cause the disease proceeds, is important; and, thus, the changes occurring in the prostate of children, are very different from those found in old men: in the former we find atrophy, or incomplete development, in the latter, enlargements, excrescences, and other signs of prolonged excessive nutrition. Among *adults* chronic swellings are rare, the maladies then taking on a more special character, usually in combination with affection of the vesiculæ seminales, or other acute malady of the genito-urinary organs, especially retention of urine. The atrophy of the gland in *children* is especially found when the bladder performs its functions badly, and there is much enuresis at night, in which cases the genital organs are slowly and imperfectly developed. Severe prostatic lesions have however followed cystotomy even in early life.

*Treatment.*—The author enters most fully into this subject, and we had prepared an analysis of his interesting observations upon the mode and difficulties of catheterism in these cases, and the treatment of the disease in its most simple form, and under its various complications; but we find it would occupy too much room, and we will therefore substitute the author's resumé.

" The treatment of the enlarged prostate, then, varies, not only in reference to its own severity, but also in respect to the influence it exerts upon the functions of other organs, and especially upon the expulsion of urine. The re-establishment of the free expulsion of this fluid is of predominant importance. But there are some important distinctions to be made.

1. When the entire or partial swelling of the gland is inconsiderable, and the genito-urinary functions have suffered but slightly from the irritation of the mucous membrane lining the prostatic portion of the urethra, it sometimes suffices to regulate the digestive organs, to relieve the constipation so common in these cases, to prescribe a mild regimen, diluent drinks, hip baths, and emollient glysters, and to remove any obstacle calculated to impede the expulsion of the urine, to obtain a relief of the slight attack to which the patient has been subjected. This is the treatment we can apply at the very commencement of the affection, but it is rare for a patient to have, at this stage of the complaint, good sense enough to apply for assistance.

2. When these means are not entirely successful, and especially if they prove useless, we must forthwith explore the condition of the urethra. Here, as in the case of simple neuralgia of the neck of the bladder, the passage of the instrument along the mucous membrane of the urethra, informs us of the degree of irritation present. If the sensibility of the canal be not excessive, and connected with but a trifling lesion, the instrument may perhaps cause a salutary counter-irritation, the symptoms may diminish, and shortly cease altogether. All that remains then is to consolidate the relief by adopting the means already recommended.

3. But, if the irritation produced be considerable, and connected with some important lesion, the patient may suffer more than heretofore for some days after the exploration. Having alleviated this temporary aggravation of suffering, the morbid sensibility of the urethra, whence it arose, must be diminished. Soft bougies, introduced with great care, and retained *in situ* from two to five minutes, form our most certain means of treatment. From eight to twelve applications, and sometimes less, suffice to allay the irritability of the canal, and to dispose it for the reception of the instruments which may be necessary for the entire cure. From the mere fact of the diminution of the sensibility of the urethra, the patient perceives a notable improvement to have resulted, which, commencing after the third or fourth introduction of the instrument, continues permanent. Frequently it has proved sufficient to continue these means for some time, and to combine with them attention to the general treatment, to procure the re-establishment of health, in a manner that could never have been anticipated. Even if this result does not follow, we have only lost a little time, and at least have exposed the patient to no hazardous treatment.

4. There are cases, however, wherein these means do not succeed, or the benefit derived is only temporary. The position of the practitioner now often becomes one of difficulty, as the symptoms take on a more active form. Moreover, his difficulties imprison him as it were in a circle, for, the patient cannot urine without a catheter, and the passage of this over the neck of the bladder reproduces all the painful symptoms. We may say now that all our efforts become limited to the surgical treatment of the symptoms as they arise, varying our means according to the urgency of the case, for we know of no mode of obtaining the resolution of an enlarged prostate, especially if it have passed into a state of induration. Our office is confined to preventing it as far as possible from abridging the life of the sufferer, or rendering the remainder of his days a continued course of suffering."

*Errors in treating enlarged prostrate* have arisen from want of experience, whence this affection has been supposed to exist where mere neuralgia or atony of the bladder were actually present, and the means used for the relief of these, have been afterwards applied to real cases of the disease, thus encreasing the number of inoperative remedies. So, too, attention has frequently been turned rather to the attendant disury or strangury than to the diseased state originating them. Owing to the supposed mere mechanical operation of instruments, in relieving retention of urine, these have been frequently used too large, and left in too long, exerting by far too much pressure on the parts with which they were in contact, and producing inflammation, ulceration, or perforation, with fatal re-action. The removal of an instrument as soon as any serious symptom occurs, and its cautious re-introduction only when this subsides, is often attended with the happiest effect. The use of *caustic* requires great caution. It may be sometimes slightly applied, when the prostatic affection is accompanied by chronic phlegmasia, or obstinate neuralgia, or when slight excrescences exist at the commencement of the affection. The influence of this substance has been much exaggerated, as it can only act by changing the condition of the vitality of the parts, which may often be effected by other means. Its application is usually too forcible. It always succeeds best after the use of the soft bougie, and the author limits himself to two or three applications.

CHAP. 2.—*Suppuration of the Prostate.*

This may occur with or without preliminary engorgement, its usual source being a phlegmasia of the mucous membrane, brought on by retention of urine, or other irritating cause. Abscess is recognized by no peculiar symptoms, and is often only discovered after death. Sometimes, collections of pus, formed in the substance of the gland, may be felt from the rectum, and these sometimes burst into the urethra or the rectum, and, as soon as the collection can be discovered to exist, an incision should be made into it. The violent use of instruments may prove a cause.

CHAP. 3.—*Ulceration.*

The greater portion of wounds and injuries of the prostate heal with facility, but in some cases these degenerate into destructive ulcers, which should be rather referred to a peculiar diathesis, than to the irritating qualities of the urine. The diagnosis is uncertain, and we have no means of arresting the progress of these fortunately rare cases.

CHAP. 4.—*Atrophy.*

Suppuration will sometimes reduce the gland to a mere hard kernel. The author, in his work upon calculous affections, has given several examples of the presence of a calculus in the bladder having prevented the development of the prostate, so that, it seems, sometimes, to have almost disappeared, while, at other times, even when stone did not exist, the gland has been perceived with difficulty from the rectum. The exact condition of the part is sometimes of difficult recognition, as there are no special symptoms, and local examination gives mere negative signs. Considered by itself, atrophy is not an alarming affection, but the functions of the bladder become more or less disordered, and there is usually defect or loss of genital power.

Chap. 5.—*Cancer.*

The affection usually termed cancer may be merely an inordinate degree of induration, which may exist to such an extent as to grate against the scalpel when divided. Cancer, properly so called, is so rare that Cruveilheir says he has never seen a case. It does however occur, though rarely.

Chap. 6.—*Prostatic Calculi.*

Calculi may be found in the substance of the prostate, in its ducts, or their orifices, and in the portion of the urethra traversing the gland, varying in colour, form, and consistence. Although occasionally vast numbers have been found, they are usually few in quantity, and small in size. They may often be detected, by passing a sound into the bladder, and a finger into the rectum simultaneously, or, when they project into the urethra, by taking an impression by means of the wax bougie. In many cases the only sign during life has been a tumefaction of the gland, and the diagnosis when complications exist is often difficult.

### IV. Diseases of the Body of the Bladder.

Chap. 1.—*Fungus and Polypus of the Bladder.*

Though not exclusively, these tumors are usually situated in the vicinity of the neck, and are often confounded with the enlarged state of the middle portion of the prostate. Their form and surface, and mode of attachment are very various, their volume being usually inverse to their numbers. They are seldom found in women or children, but are common in adults suffering from stone. Though usually accompanied with a state of phlegmasia of the mucous membrane of the bladder, there may be a preternatural paleness, and very obstinate cases of neuralgia have seemed sometimes to be owing to their presence. Their symptoms are vague and unspecial even when the case is simple, and, of course, far more so when it is complicated, as it so often is, with stone or diseased prostate. Valuable information may be obtained by exploring with lithotrity instruments, but, as the author observes, these require experience and dexterity, and may give rise to much suffering. These however form the only means we can safely employ for their removal when detected.

Chap. 2.—*Cancer of the Bladder.*

The author does not consider that the bladder is specially attacked by cancer, but that some of its affections, as tumors, &c. may degenerate into cancerous ulceration, of which he has seen several examples.

CHAP. 3.—*Tumors formed within the Walls of the Bladder.*

These are easily confounded with hypertrophy. These tumors may exist as *partial thickenings of the walls of the bladder*, which project on the external surface only, and usually occupy but a very circumscribed space, though this is not always the case. Although generally only recognized after death, in some cases the tumor may be felt through the abdominal parietes or by the rectum. In other cases, which are not rare, there may be *abscess within the walls of the bladder*, when the pus may become infiltrated into the tissues, or collected into circumscribed depôts. Sometimes these project internally, at other times externally. They may be produced by external violence, retention of urine, &c. The abscess may find vent into the abdominal cavity, the rectum, at the surface of the abdomen, and other situations. The most favorable termination is when the pus escapes by the urinary passages. The diagnosis is very difficult, but, when a collection of matter can be discovered within reach, we should open it at once.

CHAP. 4.—*Tumors developed on the External Surface of the Bladder.*

These may occur in various modes, as

A. *Irregular Distention of the Bladder.*—The bladder in becoming distended often expands unequally, especially if hypertrophied, or possessing some original malformation. The tumor it forms is elongated or flattened, and is often directed towards one of the iliac fossæ. In diagnosis we must always bear in mind the shape the bladder should assume on distention in its normal condition. In youth, it forms an oval, terminating in a point towards its summit. Later, the fundus becomes more rounded, and the organ takes a more pyriform shape. In the adult, the bas-fond becomes much enlarged, and occupies afterwards a large portion of the pelvic cavity, raised up as it were by the rectum, which, when distended, forms a notable projection upon its inner surface. In woman, the organ spreads out rather laterally, than from before backwards, in which latter direction it becomes flattened, by the womb and appendages—so that in her there may be said to be two bas-fonds, distinguishable by the catheter.

B. *Sacculi (Cellules Vesicales)* are formed by the protrusion of the mucous membrane between the muscular fibres, which occurs especially when these are in a state of hypertrophy. These pouches are sometimes numerous and small, but, at others, of greater capacity than the bladder itself. When they contain only urine, their consistence is soft, but it is firmer when they contain pus, scrofulous matter, calculi, or have contracted adhesions to the surrounding tissues. They are chiefly situated towards the bas-fond and posterior wall of the bladder. Although of common occurrence, these sacculi have frequently been passed unnoticed by reason of the small apertures existing when the hypertrophy is slight. As these cavities, when large, possess little or no expulsive power, the urine remains within them, often causing the production of phlegmasia, the deposition of calculi, &c. It is often impossible to do more than suspect the existence of this affection, identical symptoms being produced by an atonic condition of the bladder.

c. *Hernia of the Bladder.*—In *man* this occurs usually at the inguinal canal, and may exist on one side only or on both, and be formed of the bladder alone, or of it and the intestine. It may be mistaken for a common hernia, hydrocele, or inguinal abscess. The absence of the ordinary tumor caused by the distended bladder, and the co-existence of a free flow of urine and the disappearance of the swelling upon pressure, form the best diagnostic marks. Catheterism is another means of preventing error; but still, when called to a patient for the first time, suffering from retention of urine together with a large hernia, great errors may be committed in deciding as to whether the tumor comprises the urinary bladder or not. Among *women*, in pregnancy, the bladder may be forced towards the perineum; and, indeed, independently of pregnancy, this is by no means rare in them, for distention of their bladder forces downwards its neck and the urethra, thrusting the vagina before it even to beyond the vulva, and rendering the passage of a catheter difficult or impossible to the most skilful practitioner. If he succeeds he is astonished at the shortening of the canal which the displacement has caused, the external and internal orifices being almost in contact.

Chap. 5.—*On the Forced Sojourn of Urine in the Bladder.*

This may occur from two causes, viz. from the loss of expulsive power by the bladder, the passages for the urine remaining free, which the author calls *stagnation*; and from the obstruction of the urinary passages, the powers of the bladder remaining normal, increased, or diminished as the case may be, which is termed *retention*. This distinction will be found to be practically useful.

A. *Stagnation of Urine.*—In its most *simple* condition this affection exists very commonly, but, owing to the slight and vague symptoms attending its early progress, it may long escape notice. Upon questioning the patient, we shall find that for some time past he has not been able to evacuate the bladder completely, and that the urine has not flown in a full jet. It is common in neuralgia of the neck of the bladder, in diseases of prostate, and commencing stricture, &c. When the bladder becomes much distended it does not present a hard tumor as in the normal condition, but gives the sensation of a collection of fluid yielding towards the iliac fossæ. The diffused and soft nature of this tumor renders its distinction from the intestines often difficult, especially if the patient is stout, or if his tissues, as often happens, be flabby and infiltrated; while, as the bladder sometimes becomes distended to an enormous size, it has been mistaken for a dropsical effusion. Dr. Civiale has met with cases in which no kind of examination was assuring as to the condition of the bladder. In spite of great distention many patients pass small quantities of water daily, thus taking off attention from the seat of mischief. Sometimes the pain is severe, at others vague, and referred to some distant part of the body. The best means of ascertaining the condition of the bladder in such cases, is to pass a catheter as soon as the patient has ceased to urine, and employing pressure on the hypogastrium, remove that portion of urine which the natural powers were unable to evacuate, and the quantity of which will furnish some index of the extent of their feebleness. The same will be learned by injecting the bladder several times with cold water, and observing the time and mode of its rejection.

In the slightest degree of this affection it is usually discovered by mere chance, and may be *treated* with great facility. Wet compresses applied to the penis, perineum, or thighs, or the shock of cold water to some part of the surface, together with avoiding the habits which had led to its production (as, *e. g.* passing urine without getting out of bed, and the not taking sufficient time to empty the bladder,) will usually suffice. But when the early symptoms, as often happens, have been neglected, the patient going on for days, months, or even years, only partially emptying his bladder at each contraction, a state of semi-paralysis and semi-distention of the organ results, and upon the occurrence of any excess such a patient is always liable to complete retention—long prior to which, however, change in the composition of the urine, and an encreased frequency of desire and difficulty to void it, will have advertized him of the increasing gravity of the complaint. Retention arising from this cause is sometimes most improperly treated by depletory measures. The first indication is the prompt employment of the catheter, and this may be required to be repeated several times daily until a more vigorous condition of the organ prevails. This is better practice usually than leaving a catheter in the bladder, producing a more rapid cure, causing less local irritability, and not destroying the urethral elasticity. For some time after apparent cure the state of the bladder must be examined from time to time, and the patient must avoid long journeys and other causes likely to provoke retention. Bed should be little indulged in, as distention is more likely to occur while in the recumbent posture, and the catheter should be used during several evenings after its discontinuance in the day. The patient is often tormented with false desires of urining, which may be distinguished from the real by the absence of the hypogastric tumor, and by the introduction of an instrument. They should be resisted as far as possible. *Injection of the bladder,* repeated three or four times a day, and with cold or tepid water, according to the degree of atony present, are excellent means of restoration of power. In more obstinate cases the irrigation of the bladder and rectum by a continued stream of fluid, or the douche, will be found useful. The author does not approve of *counter-irritation,* except when the retention is caused by an affection of the spinal marrow, nor of stimulating remedies, such as cantharides, diuretics, balsams, &c. save at least as mere accessories.

*Complicated Cases.*—The means already mentioned do not always accomplish a cure, even in the simplest cases, and for those of a more complex character various modifications may be required. Every obstacle to the free expulsion of urine must add to the existing atony of the bladder, while the diagnosis often becomes much obscured. Strictures, and other lesions of the urethra, even when very slight, neuralgia of the neck of the bladder, lesions of the prostate, lesions of the walls of the bladder, especially their sacculated condition, atony or morbid sensibility of the rectum, may each become causes of the production of atony of the bladder, or aggravate it when existing. In all diseases of the urinary organs there is often a severe influence produced upon the general health quite out of all proportion to the extent of the local lesion, occurring especially in patients of a nervous temperament, or in persons exhausted by excess of labour, &c. In such cases there is great irritability of the urethra, and excitement of the general system, and, if instruments be roughly employed, great mischief must result. Atony of the bladder is a very common occurrence in *women* under these circumstances, although it may in other cases exist obstinately even in strong constitutions. Lesions of the nervous system form a very grave complication or cause of atony of the bladder; and in all injuries of the brain and spinal marrow the condition of the bladder should be carefully investigated, even though the patient may continue to pass some urine. In affections of the spine, the bladder becomes so gradually affected sometimes, that the symptoms may quite escape the patient's notice. The partial paralysis remaining after apoplexy also produces an atonic condition of the bladder and rectum, yet evidenced by very slight symptoms until sought for, and indeed liable to be quite overlooked, as some degree of expulsive power still remains.

It must always be recollected, that frequently with atony of the bladder co-exists a state of spasm of its neck, requiring great care in the employment of instruments. Although in most cases of atony of the bladder a state of atrophy of the walls exists, yet in some cases hypertrophy is coexistent with great enlargement of the cavity of the organ. A chronic inflammatory condition of the mucous membrane of the bladder often exists, and as it may easily become aggravated into fatal cystitis, we must introduce the instruments necessary for the removal of the urine with the utmost care.

B. *Retention of Urine.*—This is a symptom producible by many different morbid conditions, and it is wrong to look upon it as an essentially distinct malady. By reason of its frequency, retention, arising from narrowing or occlusion of the urethra, is the most interesting form of the complaint. The bladder may be influenced in two different manners in these cases. It may yield to the distending power of the fluid, and a certain portion of the urine may eventually escape by regorgement when the stricture is not too close: or it may vigorously re-act against the obstacle, its power and consequent muscular development encreasing as this is great, so that a small portion of urine is usually forced through the obstacle, sufficient to afford temporary relief, and to deceive the patient. But, upon the least excess, complete retention may occur, and, although perhaps relieved by depletion, baths, &c. will return again under similar circumstances, the accompanying symptoms and constitutional irritation placing the patient in a most perilous condition. The diagnosis, however easy in ordinary cases, is very difficult in that of a contracted bladder, for in this no tumor can be felt externally and the presence of a few spoonfuls of fluid suffice to produce the symptoms. In *treating* retention precious time is often wasted by having recourse to bleeding, baths, purgatives, &c. When it comes on after excess of table, prolonged resistance to the desire to urine, &c. the catheter must be at once, but most slowly and carefully introduced, and it will be found that the irritability of the urethra and neck of the bladder, existing in these cases, is relieved by the very same means which tends to re-establish the equilibrium between the resistance of these parts, and the expulsive forces of the bladder. The contusion of the urethra by the pressure of the child's head in labour, or the passage of an instrument, may cause such a change in the vital energy of the parts as to lead to retention, and yet no means is so effectual in restoring the equilibrium as an instrument, passed, however, on account of the great irritability present, with the greatest caution. Inflammation of the bladder may arise from retention, and forms a very grave case, but with due care a catheter may be introduced, and the irritating urine abstracted. When retention is caused by very narrow stricture, great difficulty often arises in passing an instrument, and none but the experienced must attempt it. Aid may be derived here from leeches, prolonged baths, narcotics, &c. but the author especially praises the use of very small bougies, not with the view of procuring dilatation, but of causing the cessation of the spasmodic condition of the part which is the immediate cause of the retention.

c. *Indirect Consequences of Stagnation and Retention of Urine.*—(a.) *Effects on the Kidneys and Ureters.*—The mutual sympathy between the kidneys and the bladder is well known. A slight irritation of the neck of the bladder will produce a great change in the renal secretions, while the changed composition of these latter exert much effect upon the condition of the bladder. For the urine to be secreted normally by the kidney its free expulsion must be secured, and when distention of the bladder occurs the cavities of the kidneys and ureters become similarly affected, and they also employ active and injurious efforts to obtain the propulsion. The author considers that diseases of the kidney are at present too abstractedly considered, without due reference to the condition of the rest of the urinary apparatus. He has met with cases of albuminuria and diabetes insipidus dependent upon neuralgia of the neck, and consequent atony of the body of the bladder, and which have been at once cured by relieving that condition. Much of the lumbar suffering, also, said to indicate disease of the kidney, arises from functional disorder of the bladder; and the best means of preventing organic disease of the kidney will be to treat it by establishing and maintaining the normal condition of the bladder and urethra.

(b.) *Effects upon the Integuments.*—As in the case of stone, obstinate cutaneous eruptions may result from atony of the bladder, to be relieved only as the functions of this organ are re-established. In these cases the original affection has usually made serious progress.

(c.) *Effects upon the Lower Extremities.*—Many patients suffer from pains at the inner side of the legs, or sole of the foot, as they do indeed in irritation of the neck of the bladder and calculus; but these are not to be confounded with the abscesses which may be produced in various parts of the body, as a consequence of diseases of the urinary apparatus. They are usually temporary, accompanying a desire to urine, and are unattended by fever or re-action.

(d.) *Effects upon the Brain, and Pulmonary Organs.*—Persons affected with retention are very liable to congestions of these organs; and the numbers who perish from apoplexy, brought on by unavailing efforts to urine are considerable.

(e.) *Effects upon the Bladder.*—The colour of the mucous membrane is sometimes found preternaturally pale, especially when atrophy exists: at other times, it is injected to the extent of becoming brownish or violaceous. The texture may be so softened as to tear upon the slightest pressure. But this is a rare condition to be found compared with that of hypertrophy and a diminution of the cavity.

Chap. 6.—*Incontinence of Urine.*

As in the case of retention, most authors have considered this as a special malady, while, in the majority of cases, it arises from a forced sojourn of the urine in the bladder, which is itself dependent upon some anormal condition of the urinary passages:—so that the incontinence is here literally *a consequence of a consequence.*

*Diagnosis.*—This, though easy enough as to proving the mere affection to be present, becomes more difficult when we wish to decide whether it be a true and essential incontinence, or a false and sympathetic one. When the bladder possesses slight capacity a very little urine will distend it, and then a flow from regorgement or over-distention occurs, which is liable to be mistaken for paralysis of the neck of the organ. This is also the case when we can feel the distended bladder at the hypogastrium, as we may be assured by the introduction of a catheter, after which the incontinence will cease for a few hours, until the state of over-distention is re-produced. The use of the catheter, to ascertain the quantity of urine retained, and of injections to ascertain the capacity of the bladder, and its power of expulsion, can alone throw light upon the case.

*Varieties.*—(1.) *Incontinence from Paralysis of the Bladder.*—Paralysis is usually not complete until the disorders arising from atony of the bladder have long endured and become very serious. Many circumstances exert a secondary effect upon the production of the attack of incontinence, as excess at table, neglect of the calls to urine, and various causes irritating the neck of the bladder. But riding, whether on horseback, or carriage, seems to exert an especial influence in causing it in those whose bladder by neglect has become atonic, exhibiting itself at first by a few drops, and afterwards in larger quantities. In other cases the affection appears without immediate exciting cause; but the patient is not aware often, until we question him, that his present inability to retain his urine is a mere consequence of long-standing imperfection of power of voiding it. The treatment is that adapted to atony of the bladder, and is usually very tedious; while, if the incontinence have long existed, it is often incurable, depending in this case, however, frequently, upon some cerebral or spinal lesion. If caution be not adopted in treating these cases, cystitis, hæmaturia, and other alarming consequences may result, and this has often been the case when injections of irritating substances have been employed. (2.) *Incontinence consequent upon Retention.*—Some patients, after suffering all the agonies of retention, fall into a species of collapse, during which the urine escapes drop by drop, involuntarily however, the bladder having ceased to contract. This case is, however, not quite identical with the last. When atony has preceded the incontinence it is a more serious affair, for the paralysis has been long coming on, and has at last become complete: while, in most retentions, the exhausted bladder usually undergoes a mere suspension of its contractility. In the one case, too, the urine flows continuously, unurged by any propelling force, in the other it is interrupted, occurring only drop by drop, of which the patient is aware, and often endeavours to aid the flow by voluntary exertions. In fact, here incontinence, literally speaking, does not exist, for the functions of the bladder are yet capable of propelling the urine, could the passage be freed from obstacle. If the case be not relieved, however, true paralysis succeeds, and the urine flows away unknown to the patient. The case is serious in proportion to the difficulty of removing the obstruction to the flow of urine. Sometimes the affection comes on insidiously, as, since some urine is expelled, and the patient ceases to suffer the urgent symptoms of retention, he delays obtaining the only means of relief—instrumental intervention. (3.) *Incontinence after contusion, or forcible dilatation of the Urethra.*—This is not rare after accidents, operations conducted through the urethra, and accouchement. The urethra may be greatly dilated by the efforts of nature, without the production of incontinence, as is seen in the expulsion of large calculi in women; but a dilatation produced suddenly and artificially, though of much less extent, causes it. Dilatation of the male urethra, for the removal of calculi, or the cure of stricture, may also cause incontinence; but the author denies that this occurs, as has been said, after lithotrity, since no distention is therein employed. The case must be treated by the means adapted to retention. If the urethra have lost much of its elasticity, the prolonged use of the douche, and friction of the surrounding parts, and, if the neck of the bladder have suffered, and resists other means, a few light applications of caustic, will be useful. (4.) *Incontinence after prostatic lesion, Neuralgia of the Neck, and serious affections of the Body of the Bladder.*—These affections must have proceeded to an advanced stage to produce this effect: but mere excitement of the lining mucous membrane will suffice to paralyse, to a certain extent, the contractility of the neck of the bladder. In these cases, depletory and anodyne means may require to be combined with the local procedures. (5.) *In Calculous Affections* it is not unfrequent, but certainly does not depend, as has been stated, upon a stone becoming in part engaged within the neck of the bladder. Sometimes it results from the exhaustion of the bladder after prolonged efforts, but usually from the continued irritation of the neck of the bladder, kept up by the foreign body. (6.) *Incontinence without distention of the Bladder.*—In this case, sometimes the neck of the bladder is relaxed and paralysed, or the body of the viscus may continue in a condition of permanent contraction strong enough to overcome the resistance of the neck. In cystitis, and sometimes in neuralgia, the patient may find even the contact of a few drops of urine so insupportable as to endeavour to expel it every few minutes, without which it would escape of its own accord. Analogous to the action of the sphincter ani in diarrhœa, the orifice of the urethra opens to permit the urine to pass, rather than closes upon it, the bladder still performing its functions but too actively. Our treatment must be directed to the relief of the condition of the phlegmasia, and these cases often assume a very grave aspect. In a small proportion of cases the neck of the bladder, and muscles of the perineum are attacked with paralysis, and the body of the organ possesses but a feeble degree of energy. No contractions follow repeated injection. Such cases are very serious, and often depend upon a cerebro-spinal lesion, or general failure of the constitution. Occasionally, cases are met with, in which, from organic disease, the neck of the bladder is kept in a rigid, scirrhus, and a constantly patent condition. More rarely we find the hypertrophied bladder, associated with a want of contractility of the neck, so that the organ unable to contain more than a table-spoonful or two, the surplus is continually escaping. We cannot here distend the cavity by forced injections, but temporary relief has followed the use of warm mucilaginous injections, and the employment of opium by the rectum. (8.) *Incontinence from a Dilatation of the deep portion of the Urethra.*—There are persons, who, after having expelled a jet of urine, find the remainder come away drop by drop for a long while after, so as to render precautions of dress necessary. This may depend upon prostatic lesion, stricture, and also upon a dilated condition of the deep-seated portion of the urethra; forming a kind of pouch in which urine, and sometimes gravel, becomes accumulated. Cold applications and douches to the perineum &c. glysters, and the injection of small quantities of water into the membranous part of the urethra are required, but it is long before the urethral walls re-acquire their lost tone. These pouches, depending usually upon the presence of organic stricture, or obstinate spasm of the urethra, our attention must be directed to the relief in the first instance of such conditions.

*Incontinence of Urine in Women.*—Incontinence arising from a paralytic condition of the neck of the bladder, is common in women, but, owing to their modesty preventing early application for relief, the affection is usually well established before the practitioner sees the case, and thus, although the application of instruments is easier in them than in men, the disease often assumes a graver aspect. It is an error to suppose that the bladder of women retains injections less easily than that of men, and caustic, when required in obstinate cases, is more easily applied in women, and succeeds better with them than with men.

*Incontinence in Children.*—In childhood, the functions of the bladder are performed very irregularly. Children urine abundantly, but it seems frequently to flow without producing any sensation. In general, debility of constitution, or irregular development have much to do with prolonging the affection, which, from being neglected at first, often acquires great obstinacy. The author is convinced that, in most cases, it depends originally upon an atony of the bladder, allowing large accumulations of urine, and accompanied with irritability of the neck of the bladder. By relieving these morbid conditions, the disease has often been cured. In fact, it results from a state of regorgement or reflux from over-distention, and it is rare indeed to find incontinence proceeding in children solely from a relaxed condition of the neck of the bladder. Although a disposition to this affection seems more common in some families than in others, it cannot be considered as hereditary. In many cases the malady terminates spontaneously between the years of one and five. In others we must be guided by the principles of treatment already alluded to. It is, however, at this age to the general treatment we must chiefly look, local measures being quite secondary at the commencement. Yet, when the disease has become obstinate, the catheter, injections, or even caustic may be required. There is a case which is mistaken for incontinence, viz. where, in fact, the *bladder performs its function with vigour*, the child propelling the urine in a good stream during sleep. The child should be awoke two or three times in the night to urine, and it is to this case moral correctives are to be applied.

*Incontinence in Adults and the Aged.*—So rare is essential incontinence from a paralysis of the neck of the bladder in the *adult*, that Civiale has only seen two cases. At this period of life, too, even secondary incontinence is a less frequent and less serious affection, as the maladies with which it is connected are usually curable. *Old men* are very liable to incontinence, but it is usually the effect of regorgement, and not as supposed from a diminished extensibility of the bladder. It is towards the close of life, indeed, we find those lesions of the urinary apparatus especially to prevail, which provoke a stagnation of urine, leading to incontinence. Primary incontinence only occurs in a few cases, in which an indurated condition of the neck of the bladder seems to have destroyed all its contractility, allowing the urine to escape as it arrives in the bladder; or in some others in which a very hypertrophied state of the walls is joined to a very diminished capacity of the cavity.

*Treatment.*—The author has already considered this as applied to the chief varieties, and now adds a few general observations. *Injection and irrigation* of the bladder are most important remedies, and water, gradually reduced to as cold a temperature as can be borne, seems to be the best fluid. The quantity thrown in must be moderately large, but in all our proceedings we must be careful not to excite inflammatory re-action. When injections are inadmissible, as they often are, (*e. g.* in advanced prostatic disease) or to aid them when they are not so, a catheter may be often left in the bladder with advantage. Cold local or general baths and douches are often useful, especially at the sea-side. In other cases warm or medicated baths may be required. Revulsives must not be neglected, especially when the incontinence is connected with lesion of the spinal marrow, thus, a succession of blisters may be applied to the sacrum, hypogastrium, and thighs, and tartar emetic ointment is often useful; but the former must not be employed when a very contracted state of the bladder, or irritability of its neck exists. No reliance should be placed upon the various empirical remedies. When all means of cure fail, we must have recourse to mere palliation, by the employment of mechanical inventions for collecting the urine as it flows, or preventing its egress externally.

### CHAP. 8.—*Hæmaturia.*

This is merely a consecutive effect of various maladies.

*Diagnosis.*—The determination of the existence of blood in the urine is usually not difficult, although some articles of diet, especially beet-root, produce a very similar colour. When in any quantity the blood forms into coagula, which however have a very different appearance to those formed by blood out of the bladder. The source of the blood may be the urethra, bladder or kidney. Bleeding from the *urethra* may occur in gonorrhœa or chordee, or follow excess of coition, introduction of instruments, use of caustic, extraction of calculi, &c. It may occur in quantities sufficient to excite inquietude, but flows independently of the urine, and is not accompanied by a sense of fullness and irritability of the bladder. When the deep-seated portion of the urethra is the part affected, the source of the blood often becomes a matter of obscurity. When the hæmorrhage proceeds from the *bladder*, it combines with the urine, when it exists in large quantities, but otherwise forms into black clots. The blood may proceed from tumors or other anormal growths, or from a phlegmasia, arising from excessive distention of the bladder, when it would not seem to exhale from any one part in particular, but from the whole surface. When the bladder contracts upon a large calculus it may bleed at any part, but when the stone is small the hæmorrhage usually proceeds from the neck. The bleeding is judged to proceed from the *kidneys* and *ureters* when no cause for its existence can be discovered in the other portions of the apparatus, or when some injury has been inflicted upon the lumbar region: but the case is frequently purely conjectural, and hæmorrhage arising in consequence of over-distention of the bladder is frequently attributed to the kidney, and as our means of exploration have become more accurate, the number of renal hæmorrhages have decreased.

*Varieties.*—(1). *Hæmaturia in connection with Stricture, Neuralgia, and Atony of the Bladder.*—A neuralgia of the neck of the bladder, accompanied by little or no stricture, but by a mere rigidity of the urethra, may give rise to an exhalation of blood. Indeed, when the urine is retained in the bladder from any cause, it may become sanguinolent, and many patients fall victims to the affection whom a prompt use of the catheter would have saved. Distention of the bladder, especially in the aged, is the commonest of all causes of hæmaturia. (2.) *Hæmaturia in Calculous Affections.* Contrary to what most authors state, stones of an irregular exterior do not give rise to hæmorrhage oftener than those whose surface is smooth and polished. Hæmaturia in these cases is usually not very abundant, unless some organic lesion of the bladder be present. (3.) *Hæmaturia from Organic Lesion.* The appearance of hæmorrhage in fungus, ulceration, tumors, &c. of the bladder has frequently been the cause of the patient's first applying for medical aid for an affection hopelessly advanced. The symptoms accompanying it sometimes resemble those of stone. Many persons having various affections of the urinary passages, are very liable to hæmorrhage after journeys. (4.) *Hæmaturia in Cystitis.* This proves the inflammatory action has reached a great height. To this cause, and inflammation excited in the kidneys, various anomalous forms of hæmorrhage are to be referred. Articles of diet, as truffles, according to the author, produce it. Stimulating injections for incontinence of urine, and both the external and internal use of cantharides, have produced the same effect. (5.) *Hæmaturia from Violence.* When injuries to the perineum, riding on horseback, &c. produce this affection, some lesion of the urinary apparatus has usually predisposed to it. (6.) *Hæmaturia from Atmospheric Causes.* The army surgeons attributed the frequent occurrence of this affection in Egypt to the high temperature. Dubois, the celebrated surgeon, was obliged to return to France on account of hæmaturia. In many tropical regions the affection is endemic, and is frequently accompanied by gravel. Feeble persons sometimes lose an ounce or two of blood daily for several years without apparent effect upon the health. When the affection has become obstinate, mere change of climate will not relieve it. (7.) *Periodical Hæmaturiæ.* In some rare cases, a congested state of the urinary passages gives rise to a periodical flow of blood, especially in women, after the critical age, and in men who are liable to hæmorrhoids. Without denying that these bleedings may be sometimes metastatic and succedaneous, the author blames the generalizing into a law a few exceptions, and thus neglecting an exact exploration of the state of the urinary organs prior and subsequent to the flow of blood. These hæmaturiæ are usually obstinate, but produce remarkably few ill effects. Every variety as to the appearance, quantity, and progress of the hæmorrhage may occur. (9.) *Critical Hæmaturiæ.* The author expresses his doubts as to the existence of these, and many of the affections, as atony of the bladder, lesions of the walls and neck, whose resolution the hæmaturia is said to have procured, are the very conditions which have led to its production.

*Frequency as to Age.*—The voiding of blood is very seldom found in children, even when calculi are present, and in them explorations of the urethra and bladder are seldom followed by bleeding. It is in *old age*, when the bladder so frequently becomes over-distended, that the affection especially prevails, a condition of the bladder very favourable to it, conjoined with the determination of blood towards the pelvis, which occurs in advanced life.

*Prognosis.*—This depends chiefly upon the gravity of the cause producing the malady. Hæmaturia, dependent upon a mere passing distention of the bladder, is soon capable of relief; but Dr. C. considers the opinion that the voiding blood after suppressed discharges is beneficial rests upon slight foundation. In some cases the mere abundance of blood has proved fatal, but, generally speaking, danger is by no means proportionate to the quantity lost: for, some patients, of apparently no great resources, lose large quantities with little enfeebling effect, while others have become entirely cast down by very slight discharges.

*Treatment.*—After reviewing the various means employed by authors for the treatment of this affection, as bleeding, astringents, injections, &c. the author observes these may become in different cases useful auxiliaries; but, employed exclusively, without attacking the evil at its root, they can seldom succeed, and may prove hurtful by entailing the loss of precious time. The extraordinary farrago of medicines which have been proposed for the relief of the disease, proves the ignorance which has prevailed respecting its nature.

The proper treatment is simple, for as the affection almost always arises from an over-distended bladder, our object should be to relieve and prevent the recurrence of such distention, and when applied to before the general system has suffered from the disease, our means of effecting this do not often fail us. The treatment in fact appropriate for atony of the bladder is here indicated. The urine is to be carefully, slowly, and with pauses removed. The manner of its flowing will indicate the degree of contractile power remaining, and the necessity of injection. The source of the hæmorrhage, however, is sometimes not discoverable, and the treatment unsuccessful or at all events very tedious, during which the general health may give way. Periodic flows of blood must be merely watched and met with hygienic precautions, and no active efforts at their suppression, especially in the aged, must be made unless the hæmorrhage reach a dangerous extent. These cases, said to be so common, are in fact rare, and the examples the author has met with have not occurred in the feeble, but in the robust and well-nourished. There are some good directions for the management of catheterism when the bladder contains coagula.

CHAP. 9.—*Catarrh of the Bladder.*

There is no affection of the bladder more frequently met with, and none upon which definite ideas of treatment are less entertained. It is a phlegmasia (usually chronic) of the internal surface of the bladder, producing a special character of the urine, arising from the presence of the anormal secretion of the mucous membrane. The secretions may be *mucous*, presenting every variety in consistency, quantity, and colour; sometimes accompanied by peculiar sensations during their expulsion, sometimes not: generally inodorous, but occasionally far more fetid than even ammoniacal urine. Again, the products of the mucous follicles may be *puriform*, or nearly resembling pus, and usually mixed with mucus. They may be distinguished from true pus by the urine which contains them reddening turnsole, while urine containing pus possesses alkaline properties: pus, too, exposed to the flame of a candle will inflame, while mucus only chars. If, also, the urine be decanted, and cold water poured upon the deposits,—these, when mucous, will be raised in little masses or filaments, swimming on the surface of the fluid without mingling with, and scarcely colouring it: while, pus commingles with the water, colours it of a yellowish-white, much resembling the turbid urine when first voided, or when stirred prior to decanting it. Lastly, the discharges may be truly *purulent*, the urine becoming alkaline, or at least neutral. Alkalescence of the urine, however, may occur in a healthy state of the bladder, or under the influence of several diseases. When the deposits are truly purulent, the general health is usually much disordered, and the presence of some serious organic lesion is to be feared. The symptoms of catarrh which the author cites have nothing diagnostic, being similar to those arising from many other of the affections of the urinary apparatus. If left to itself the disease usually proves eventually fatal; but it is often successfully treated when commenced with early, and due attention paid to the nature of its cause. When death does occur, it is less due to the phlegmasia itself than to secondary lesions.

Instead of a chronic phlegmasia, acute *cystitis* may exist, and which may arise from local or general causes, and may be idiopathic or symptomatic of affections of the womb, rectum, &c. The distressing symptoms here present, the author considers depend so much upon the presence of urine, that if an instrument could early in the case be introduced into and maintained in the bladder, they would in a great measure disappear. The distention of the bladder, producing first hæmaturia, and then cystitis, may have been going on for a very long period. It, however, often occurs in hypertrophy of the bladder, producing still more serious symptoms. The combination of cystitis and retention is indeed an alarming one. There is often violent spasmodic action simultaneously present. When the case is purely accidental and properly treated it is manageable, but, when it supervenes on preceding chronic phlegmasia, it may give rise first to violent re-action, and then to alarming prostration. The distinction between cystitis, and the merely irritable bladder is not always easy, especially when it is only partial. It usually commences at the trigone, where, too, is the seat of the most violent symptoms throughout.

*Post-mortem Appearances.*—In chronic catarrh, the mucous membrane may be found livid, soft, swollen, and injected to a greater or less extent, consisting in the early stages of mere patches here and there. In some old catarrhs, whose symptoms have not been urgent, it is found præternaturally pale. Frequently there is a greyish pseudo-membrane, especially at the neck, of a considerable thickness, giving the bladder the appearance of having been macerated. Ulceration in every variety may occur, especially when cystitis has supervened, producing even perforations and fistulous communications with other organs. Suppuration also occurs in violent inflammation, the walls of the bladder being infiltrated with pus. The termination in gangrene occurs chiefly in subjects affected with stone, but may do so independently; but it must not be believed to exist merely because the mucous membrane is found of a livid or blackish colour, which may occur when the inflammatory symptoms possess little intensity. The submucous tissue may become so indurated and thickened as to be mistaken for scirrhus, and this occurs sometimes when the fleshy fibres have undergone no alteration; at others, these acquire immense development, forming marked hypertrophy; and in other cases, the whole of the vesical tissues become blended in one undistinguishable mass of disease. Instead of induration, ramollissement is sometimes found, accompanied with infiltration, the tissues being bathed in a reddish purulent sanies. The thickness the walls of the bladder acquire in consequence of some of these changes, enables us to feel it as a projecting tumor above the pubes, even when it contains no urine, under which circumstances, also, its capacity is usually so much diminished as to be able to contain merely a few spoonsful. The *urethra* sometimes exhibits also marks of inflammation, so that no line of distinction exists between its mucous surface and that of the bladder. It is rare, however, to find inflammatory action propagated from the bladder to the *ureters*. The *kidneys* suffer often from protracted catarrh, becoming inflamed, suppurating and dilated, and in this case the mucous membrane of the ureters is inflamed and their cavity filled with pus. Disease of the kidney, indeed, as in the majority of the affections of the urinary apparatus, proves the immediate cause of death in most cases.

*Varieties.*—Various affections of the genito-urinary apparatus may give rise to the phlegmasia of the mucous membrane producing catarrh of the bladder.

(1.) *Catarrh in Neuralgia of the Urethra and Neck of the Bladder.* Here the affection is of slight extent and little consequence, and need not specially fix our attention farther than to indicate to us, that the neuralgic irritation has acquired a certain degree of intensity, and is in course of propagation from the neck to the body of the bladder. Our treatment of the original affection must not be modified in reference to the catarrh, unless this has been already ill-treated, or the entire case much neglected.

(2.) *In Stricture and Induration of the Urethral Parieties.* Although in treating an ordinary stricture the catarrh will be relieved as the stricture is cured, yet, in one that has been long neglected, the catarrh may become considerable and obstinate, and is liable at any time to be converted into a fatal cystitis. The treatment of the complication of a narrow stricture, retention, and cystitis is exceedingly difficult, for relief to the stricture can by no means be suddenly given, while the passing an instrument for the mere temporary removal of the retention frequently causes great agony and encreased inflammation, so that, in spite of our wishes, we often permit the acrid urine to continue distending the irritable walls of the inflamed bladder. And yet difficult as it is of accomplishment, the relief of the retention is of the first importance, it being usually the primary cause of the cystitis. In cases where there is very slight stricture, or merely the loss of the suppleness of the urethral walls, attention may be easily taken off from this condition of the canal, which is the cause, and turned to the mere catarrh, which is only the effect. The soft bougie forms here an effectual means of treatment. A catarrh also of an obstinate kind may depend upon a too narrow or obstructed orifice of the urethra and cease as soon as this is liberated.

(3.) *In Disease of Prostate and Neck of the Bladder.* These important lesions may induce catarrh by the propagation of phlegmasia from the neck to the body of the bladder, but it probably usually proceeds from the distention of the bladder they produce by the obstruction they occasion to the free egress of the urine. The affection is frequently insidious in its progress, which has far advanced before the patient applies for aid, seeking only for relief from the discharge, and unaware of the important lesions whence it proceeds. Too often indeed we see the case when mere palliation is all that is in our power. A careful exploration, frequently occasioning great pain, is the only means we have of arriving at the exact condition of the parts; and we shall find the expulsive powers of the bladder are defective, and that, after the patient has ceased to urine, a portion of muddy fluid may be removed by the catheter. Catarrh proceeding from affections of the deep-seated portion of the urethra, when complicated with affections of the *genital organs,* offers a very serious case, occurring usually in patients of a very irritable temperament. When of old-standing, it is often hopeless, and the most persevering treatment by the bougie or caustic is too often unavailing.

(4.) *From Fungous and other Tumors of the Bladder.* The discharge is of course a mere effect, indicating by its abundance the progress of the affection. In these cases, it is usually very thick, containing portions of the diseased structure, and attended with horrid fœtor and frequent hæmaturia. ' Sacculi of the bladder may also be affected with violent phlegmasia, and giving rise to abundant catarrh.

(5.) *From Urinary Calculi.*—Under the influence of the catarrh, calculi may be formed, while, when they exist, they serve to aggravate the catarrh. Renal calculi rarely produce it, or only after they have long existed. The physical characters of the stone in the bladder, and constitutional peculiarities of the patient give rise to great varieties: for while in the last stages of calculous disease no catarrh may exist, in others, it is found with the earliest appearance of the stone. It may occasionally persist after the removal of the stone, and aid in its reproduction. It usually depends upon the atonic condition of the bladder, but at other times upon local injuries inflicted by the stone or instruments.

(6.) *Catarrh from Hypertrophy.*—Hypertrophy may be either a cause or consequence of the catarrh, of which we judge by the manner in which the patient urined prior to the appearance of the discharge. The suffering is great and the case may easily pass into one of cystitis, accompanied with dreadful anguish.

(7.) *Catarrh from Atony and Incomplete Paralysis.*—More than one-half the catarrhs are the direct consequence of this condition. It is here occasioned by the unavailing efforts of the bladder at complete evacuation, or by the irritating effects of the stagnated urine. The affection comes on so insidiously, that it may have far advanced, and the constitution received a severe shock before the patient applies for aid. Although an examination by the catheter can alone reveal to us the nature and extent of the disease, its employment is not inculcated in books. The various complications of neuralgia, hæmaturia, (which is frequent in the aged,) &c. require appropriate modifications of treatment. In very obstinate cases slight applications of caustic, or the injection of a weak nitrate of silver lotion are often useful. A host of empirical remedies have been tried, and proving sometimes useful by chance, have been erected into a general plan of treatment.

(8.) *Catarrh from Indirect Causes.*—There are a few cases which we cannot always refer to direct causes, but too much importance has been attached to those of an indirect nature, even when the former are detectible, and the treatment has too frequently been directed in reference to these alone. Thus a catarrh of the bladder may occur after the cure of various cutaneous affections, and especially after diseases of the mucous membranes, but, these will almost always be found, connected with *atony of the bladder.* So, too, in many old men, attacks of gout and vesical catarrh are simultaneous or successive, and, in these subjects, the natural disposition to atony of the bladder is strengthened by the repose the malady exacts. By the arrest of transpiration and exanthematæ, which has often preceded catarrh, many authors have explained its occurrence, and Dupuytren accounted for its prevalence among the aged by the harsh condition of the integuments which prevails at that period of life. In all similar cases that our author has seen, he has found irritation at the neck of the bladder, atony of that organ, or some such predisposing cause; but he admits the important aid derived in treatment from the due regulation of the cutaneous functions. The frequent occurrence of the affection in the sedentary and studious is another proof of the correctness of this explanation of the cause.

*Catarrh of the Bladder in Women.*—Little attention has been directed to the occurrence of this affection in women, as they are far less liable than men to lesions of the urinary apparatus, and complain less frequently even when afflicted with them. Yet it is common enough among them, at all ages, and in all conditions, and frequently assumes a very obstinate character. Not only is it found in girls liable to incontinence, but in others in apparent health, but who, upon examination, will be found to have irritability of the neck of the bladder and atony of the organ. It is observed also in difficult early menstruation. The habit of drinking a too small quantity of fluid, and of *resisting the desire to urine*, are prominent causes. Among new-married women the abuse of coition, or gestation may produce the catarrh in those predisposed, but the case is rare, and only becomes serious by neglect. The worst cases occur in women of nervous temperament, who abandon themselves to unlimited sexual pleasures and spirit drinking.

*In Children.*—It is common in its chronic condition in children, and even may occur in its acute form, which cannot surprise us, when we consider the atonic condition of the bladder at this period of life. It occurs especially in scrofulous and ricketty subjects. It is very insidious in its progress; and years may elapse before the discharge becomes purulent, and the constitution gives way. It is easily treated at the commencement by bougies and injections, but then we rarely see it at such stage; and afterwards the irritability of the canal presents many difficulties. Worms, and other irritation of the rectum exasperate the affection.

*Treatment.*—(1.) *Antiphlogistics.*—We must not look upon this as a mere ordinary phlegmasia, to be met with antiphlogistics. These are required in the acute but rare form, but in ordinary cases, although not to be absolutely neglected, they are merely secondary, our principal object being to secure a due expulsion of the urine. (2.) *Narcotics.*—These are of utility introduced by the mouth, rectum, or skin, in order to relieve the neuralgic and other complication: so as to prepare the parts for more direct measures. Carried to excess, they induce constipation and debility, and favour the production of atony of the bladder. (3.) *Balsams.*—The author criticises the empirical manner in which Dupuytren employed turpentine, without any reference as to whether organic lesions existed or not. In most of his cases he was obliged to suspend it by reason of the exasperation of the phlegmasia it excited. Mere temporary amelioration has often been registered as a cure, and even where the latter really did occur, it may have been due in part to the improved regimen and circumstances under which the patient was placed. When the catarrh depends upon an excited state of the neck of the bladder, and constipation coexists, it may act usefully through the medium of the alimentary canal. Moreover, in some slow, chronic inflammations, its stimulating effects seem to have been beneficial: and towards the end of a long course of treatment, where the original causes of the disease have been removed or diminished, it may exert an influence in changing the condition of the vitality of the parts: but, for this purpose, injections and glysters of cold water, and aperients are usually to be preferred. (4.) *Revulsives.*—Blisters applied to various localities have been recommended, but the author has not derived much benefit from their employment, and they often produce strangury. Antimonial ointment applied to the hypogastrium or sacrum, has succeeded better. He uses neither the seton or cautery. (5.) *Injections of the Bladder.*—These are very useful in removing the deposits, and in restoring the natural sensibility and contractility of the bladder. Pure water, at first tepid, and afterwards employed at a temperature low in proportion as the atony is considerable, is to be thrown in several times daily, in sufficient quantity to excite the desire of expulsion. The number of times may be diminished, and the temperature of the water increased, in proportion as the contractile power of the bladder is restored. When made carefully and slowly, these injections cause little or no pain, and are attended with the most satisfactory results. Various medicated injections have been indiscriminately recommended; the nitrate of silver, however, is a powerful modifier of perverted sensibility. Injections of water must precede its use, and the solution employed must be very weak, When sacculi exist in the bladder, the water injections should be employed after the use of the nitrate, so as to secure its proper evacuation from these receptacles. (6.) *Solid Nitrate of Silver.*—This should be limited to cases which only implicate the neck of the bladder, and when great irritability of this part, conjoined with hæmorrhage, has prevailed, it is often useful, applied however, in the most delicate manner. (7.) *Warm Sulphureous Mineral Waters* seem, when the case has depended upon mere atony and has become obstinate, to be of great utility. When, however, neuralgia of the neck and tumefaction of the prostate exist, retention may follow their employment. Much of the benefit derived arises from the encreased quantity of diluent fluid the patient drinks, and on his discontinuing this, he frequently finds all his ailments reproduced.

It is seldom that so useful a *practical* work has issued from the French press of late. The author illustrates every point by abstracts of cases furnished by his own extensive practice in this branch of surgery, nor is he niggardly in referring to the experience of others, among whom our own countrymen hold a conspicuous place. The book is an able protest against the rash surgery too often witnessed in the class of affections to which it relates.

---

SIR BENJAMIN BRODIE informs us, in an advertisement, that he has introduced into the present edition the results of his later experience. He adds:—" The present volume is not very much increased in size as compared with its predecessors. Nevertheless, with the exception of the Lectures on Calculi of the Urinary Bladder and Lithotomy, there are few parts of it which remain such as they were formerly. Several errors are, I hope, corrected: some of the views which I had been led to entertain of disease are modified; and there is a considerable proportion of new matter. In the latter is included a Lecture on the Operation of Lithotrity, on which in the former editions of this work I did not feel myself competent to offer more than a few general observations. I have now ventured to discuss this new mode of treatment more at length, giving some practical instructions for the performance of the operation, which may probably be acceptable to the younger members of our profession, and to those whose minds have not yet been directed to the subject; at the same time endeavouring to assign to it what I believe to be its proper place among the appliances of surgery, and what, if I am not greatly mistaken, will be conceded to it by others, when time and experience shall have dissipated alike the prejudices of those who underrate its importance and usefulness, and of those who hold it to be more useful than it really is."

Sir Benjamin Brodie's contributions to the literature of surgery are so eminently practical, and so valuable to those who prefer facts to theories, that we have never neglected an opportunity of laying them before our readers. And we would take this opportunity of observing, that works of this description should be brought prominently forward, and held up as models to young medical men. The taste of the day would seem to be inclining too much towards compilations, learned, no doubt, and laborious, but adapted for the closet, rather than the field of practice, and better calculated to make men talk than act. In the literary productions of Sir Benjamin Brodie we find a totally different spirit. They contain the knowledge which is taken from the bedside, and can again be taken to it. There is no parade of quotations, no innumerable references to the opinions of others, no transcendental speculations, no ingenious and unsubstantial disquisitions, no extravagant views, no hunting after what is merely new, nor bigotted adhesion to what is merely old, no disposition, in fact, to deviate from that straight path of moderation and of common sense, which leads in the applied sciences, as in the common business of life, to the most successful and solid results.

What we most want in our profession to make one man's knowledge available for many, is acuteness in observing and fidelity in reporting, and both these requisites appear to exist in Sir Benjamin Brodie. Those who enjoy the pleasure of his acquaintance are aware of his quickness in appreciating the subtle indications of disease, a quickness which, probably, more than any other quality, has placed him in his proud position. And his truthfulness in relating what he has remarked, is attested by the healthy popularity of his works, and the general stability of his opinions. In giving, then, a prominent place to those opinions, and as ample a notice as possible to those works, the critic is not merely disseminating a certain quantity of truth, but he is effecting a more extensive and a higher object, in inculcating a sound and right spirit of investigation.

The present edition of Sir Benjamin Brodie's work contains some new matter, and differs so much from its predecessors as to lead us to examine it with care, and lay what is fresh before our readers. The nature of the task will preclude, of course, a circumstantial analysis, and give it somewhat of a desultory and unconnected character. This, however, is equally unavoidable and unimportant.

The first four Lectures are occupied with the consideration of

*Stricture of the Male Urethra.*

The observations on Abscess in the Perineum, and Fistula in consequence of Stricture, are more extended than before.

Sir Benjamin thus describes the ordinary course of the affection. The patient, he says, complains of more than usual difficulty in voiding his urine; but the difficulty does not amount, at least in the first instance, to an absolute retention. Perhaps he has a shivering. There is a sense of fulness in the perineum, and some degree of deep-seated induration is perceptible in one part. This gradually increases, and a tumor presents itself under the skin of the perineum, surrounded with more or less of œdematous effusion, especially into the scrotum. The skin becomes inflamed, and the fluctuation of fluid is perceptible underneath. An abscess bursts, or is opened with a lancet, and a considerable quantity of putrid pus is discharged. Here the œdema of the neighbouring part subsides. Pus continues to flow through the orifice of the abscess, and after some time it is observed that urine flows through it also. The discharge of pus diminishes, but the urine flows in larger quantity; and whenever the patient makes water, part escapes through the natural channel and part by the new opening. The abscess has evidently a communication with the urethra, behind the stricture. If you have an opportunity of dissecting the diseased parts while the abscess is recent, you find it to open into the urethra by a ragged irregular orifice. If you examine them at a later period, the orifice in the urethra is found to be smooth, regular, and rounded at the margin; the external orifice in the perineum is reduced to a narrow diameter, and is seen in the centre of a button-like projection of the skin: and the abscess itself is contracted, perhaps reduced to a narrow passage, with a smooth surface, which presents somewhat of the appearance of it being lined by a mucous membrane. We now say that the case is one of *fistula in perineo.* The whole of these phenomena are easily explained. The urethra, constantly teased by the pressure of the urine against it, ulcerates behind the stricture. If the stricture had been completely closed, as in a case of retention of urine, an extensive extravasation of urine would have immediately taken place; but under the existing circumstances this does not happen, and only a moderate quantity, perhaps not more than a few drops, dribbles into the cellular membrane, sufficient to induce inflammation and suppuration and no further local mischief. A *fistula in ano* is formed in the same manner, by ulceration of the rectum allowing the escape of a minute quantity of fæculent matter into the neighbouring textures.

Sir Benjamin goes on to remark, that sometimes the abscess in the perineum is accompanied by typhoid symptoms. If the abscess is opened, the matter that issues is putrid and urinous—if the opening is deferred the patient may die.

"I have described," continues our author, "the simplest form of the urinary abscess. But it is often more complicated. It is not always confined to the perinæum. Sometimes it makes its way forward through the upper part of the scrotum, and presents itself on the lower part of the penis, between the scrotum and the glans. At other times it burrows in the opposite direction, forming a large collection of matter in the nates, or it may burst in the groin, or in the scrotum. In one case, in which I had the opportunity of examining the body after death, I found a large abscess in front of the pubes, extending half-way towards the navel; another among the adductor muscles of the left thigh; and a third among the muscles at the upper part of the right thigh, as far outwards as the *foramen ovale* of the ischium; the periosteum having been destroyed, and the bone itself rendered carious to a considerable extent: and all these abscesses could be traced into an abscess in the perineum, communicating with the urethra behind a stricture by a small orifice. In another case which I attended with Mr. Samuel Cooper, there was a *fistula in perineo,* communicating with a large abscess of the pelvis on one side of the bladder.

I have seen a few cases in which an abscess of this kind had made its way into the rectum, forming a fistulous communication, between it and the urethra. If such communication be of a large size it is a source of great distress, as fæculent matter occasionally passes through it from the rectum into the urethra. If it be small, however, the absolute inconvenience is trifling, and the patient is rendered sensible of its existence only in consequence of a small quantity of air escaping occasionally by the urethra; and this may continue, without any further symptoms supervening, for many years." 19.

To our anatomical readers the directions taken by the abscess will be explicable by the relations of the fasciæ.

"There is one form of abscess of the perineum, which may be compared to what has been called a *blind fistula of the rectum;* the abscess having an opening into the urethra and none externally. Such an abscess may at one time be inflamed, swollen, and tender; then these symptoms may subside, but only to recur at a future period; and this state of things may continue for many years. I conclude that in these cases the abscess is formed in the usual way, by ulceration of the urethra and the infiltration of a small quantity of urine into the cellular texture; but that, when a certain quantity of matter is collected in it, it bursts into the urethra, instead of finding its way to the surface, the communication being of sufficient size to prevent any considerable accumulation of matter afterwards.

A fistula of this description is a source of inconvenience and mischief, and of nothing else. It is not so with a fistula which has an external opening. The latter answers, in some measure, the purpose of a safety-valve to the bladder, enabling the patient to void his urine even where the stricture is closed, and lessening the liability to retention. But even in this case the good is not unmixed with evil. It occasionally happens that the external orifice of the fistula becomes inflamed and swollen, or that it actually heals, and that this is followed by an accumulation of matter within, attended with many or with the whole of the symptoms which marked the first attack of the disease. And there may be even greater mischief ultimately. Mr. Vincent and myself attended a gentleman with a *fistula in perineo,* which he had neglected for many years. At last he observed that the callosity at the margin of the fistula had begun to increase; and it went on increasing so that it ultimately extended to the scrotum and penis. When we were called in we found him with a malignant tumor, affecting the perineum, scrotum, and penis, which had evidently had its origin in the fistula. He ultimately died in great distress and misery." 20.

It has appeared to us, that these abscesses in the perineum occasionally take place independently of any ulceration of the urethra, or escape of the urine. A patient has inflammatory gonorrhœa, or stricture, or both. A swelling forms in the perineum, in the course of the urethra. It is opened. The pus is not appreciably mixed with urine, nor does any urine subsequently issue from the opening. The symptoms and the course of the disorder indicate sufficiently the connexion of the abscess with the urethra, while no evidence exists of ulceration of the latter. Nor is this inconsistent with analogy. In inflammatory gonorrhœa, we meet with abscess in the corpus spongiosum, or even in the subcutaneous cellular membrane of the penis. Irritation of the rectum will give rise to suppuration in the ischio-rectal fossa, and irritation of the mucous membrane of the cæcum to abscess in the iliac fossa. Inflammation of the mucous membrane of the fauces will occasion the formation of matter in the cellular tissue, external to them, while one of the most deadly consequences of laryngitis arises in the same manner. From direct observation, then, as well as from reasoning, we are inclined to believe that, in cases of stricture, as well as in other inflammatory affections of the urethra, abscess in the perineum may occasionally form without ulceration of the walls of the canal.

*Combination of Stricture with Enlarged Prostate Gland.*—Speaking of this, Sir Benjamin remarks, that, although simple chronic enlargement of the prostate usually relieves a stricture, yet, where the disease of the prostate goes beyond a mere enlargement, and suppuration has taken place in its substance, an opposite effect is produced on the stricture; the abscess itself becoming a source of irritation, rendering the stricture more sensitive and more liable to spasm than it would have been otherwise.

No doubt persons are often said to labour under stricture in combination with enlarged prostate, when there is no stricture at all. The blunder, indeed, is not confined to the combination of the two complaints—it extends to either singly. Many persons are treated for stricture who never had any, and for enlargements of the prostate which exist only in imagination.

*Introduction of the Bougie.*—We are tempted to introduce Sir Benjamin's directions for the introduction of the bougie. "The best kind of bougie," he observes, "is that in common use, made of plaster spread on linen, and rolled up. It should be smooth on the surface, and neatly rounded at the extremity. The plaster bougie should be rubbed until it becomes warm, so that it may be moulded by the hand, and bent into the form of the urethra. Thus bent, it is much to be preferred to the elastic bougie, which is made of elastic gum on the outside and of catgut within. The latter may, it is true, be bent into any form: but it is elastic, and however you may bend it, it always regains its straight figure; and hence it is not well constructed for being passed along the curved canal of the urethra. The bougie which is used for the purpose of examining the urethra should be of a full size, that is, large enough to fill the urethra without stretching it. A small bougie may deceive you in two ways: it may pass through a stricture, and thus lead you to believe that there is no stricture, when there really is one; or it may have its point entangled in the orifice of one of the mucous follicles of the urethra, or in some accidental irregularity of the canal, and lead you into the opposite mistake of supposing that there is a stricture where none exists, If you use a bougie of the size of the urethra you are not at all liable to the first error, and you are much less liable to the second than you would be otherwise. The bougie should be cylindrical. There is no advantage in any bougie, except a very small one, being conical. A conical bougie becoming larger towards the point which is held in the hand, is likely to extend forcibly the orifice of the urethra, and to excite inflammation in it.

The existence of stricture in the anterior part of the urethra, or at its orifice, is so easily ascertained that it seems unnecessary to offer any observations on the subject. The following rules, then, are to be considered as relating especially to those cases in which it is a question whether there be or be not a stricture in the membranous part of the urethra, or in its immediate vicinity.

I generally find it best to introduce the bougie with the patient in the erect posture, keeping the extremity of it, which I hold in my right hand, close to his groin, and passing it until it will go no further in that direction; after which, by turning the instrument, I bring it horizontally forwards, and push it gently towards the bladder. If the patient has well-marked symptoms of stricture, and the bougie meets with an obstruction in some part of the urethra, you may be justified in considering this as sufficient to indicate the existence and situation of the disease. If, however, the patient has no such well-marked symptoms, you should not advance at once to the conclusion that there is a stricture because the bougie does not immediately enter the bladder. The extremity even of a large bougie may hitch in some irregularity of the mucous membrane; or if you are at all rough in the use of it, a spasm may be induced in the membranous part of the urethra, or in the muscle which surrounds it, preventing the bougie from being passed, although no such cause of obstruction exists at other times. Under these circumstances you should introduce a silver catheter, or what is better, a metallic sound, having a moderate curvature, and warmed to the temperature of the body; and it is probable that, if there be no stricture, the metallic instrument will be easily introduced, although the plaster bougie could not be introduced at all. In short, where there are no decided symptoms of stricture you ought not to adopt the opinion that a stricture exists without having made a very careful examination of the urethra. Inattention to this rule has led to many patients being subjected to a course of treatment for stricture who had never laboured under the disease."

Sir Benjamin makes some just observations on the tendency to fashion too prevalent in medicine. "There is," he says, "a fashion in diseases, or rather (to speak more properly) there is a fashion in the opinions entertained as to the prevalence of particular diseases, and when the attention of the medical profession and the public has been especially directed to a certain order of cases, such cases are almost invariably supposed to be much more common than they really are. A very few years ago it was so with respect to the disease which we have now under consideration. If a man had a troublesome gleet; if he had an indurated testicle; if he had a priapism at night; if he had a frequent inclination to void his urine; if he was impotent, or believed himself to be impotent; if the stream of urine was not perfectly cylindrical; or even if he was liable to an herpetic eruption on the prepuce; he was supposed by many surgeons to be labouring under stricture of the urethra, and was at once subjected to the unnecessary use of bougies. The number of persons who at this period were supposed to have a stricture of the urethra, and who really had no such disease, and many of whom had no disease at all, was not less than that of the young females who, at a still later period, have been the victims of another mischievous delusion, being laid up for years together on a sofa, under the supposition that they laboured under disease of the spine or hip, when in reality they suffered only from hysterical pains and spasms, which air and exercise would have cured, but which confinement and nursing, and the attendance of physicians and surgeons, have only tended to aggravate."

*Treatment of Retention from Spasmodic Stricture.*—Sir Benjamin recommends the immediate and direct recourse to mechanical means.

"Begin," he says, "by taking one of the smallest gum catheters, which has been kept for a considerable time on a curved iron wire, and which retains the curved form after the wire is withdrawn. Introduce it without the wire; and, as it approaches the stricture, turn the concavity of the catheter towards the pubes, elongating the penis at the same time by drawing it out as much as possible. It is not very improbable that it will pass through the stricture, and enter the bladder. The urine will then flow through it in a fine stream, and the patient will obtain immediate and complete relief.

If you fail with the small gum catheter, try, not a plaster, but a small catgut bougie. Let this be well made; that is, firmly twisted, nicely rounded at the extremity, and every where well polished. Observe the same rule of elongating the urethra, and it will probably enter the stricture. It is not necessary that the catgut bougie should pass on to the bladder; it is sufficient if the stricture grasps, or holds it. Let it remain in the stricture until there is a violent impulse to make water. Then withdraw the bougie, and the urine will follow it in a small stream. If the patient empties the bladder, the object is attained; but, otherwise, re-introduce the catgut bougie, or rather introduce another of the same size (for a catgut bougie which has been once used is not fit to be employed a second time); and let the patient retain this second bougie as long as he can. If the straight catgut bougie cannot be passed, you will often succeed in effecting its introduction by bending the point of it thus:—

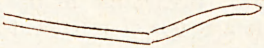

This contrivance enables you to keep the point sliding against the upper surface of the urethra, avoiding the lower part, in which the obstruction is always most perceptible, and in which the bougie is most likely to become, as it were, entangled.

Even where you have failed to relieve the patient by means of the catgut bougie, you will often succeed in introducing a silver catheter, or an elastic gum catheter mounted on a firm iron stilet, into the bladder. The catheter employed on this occasion, if the stricture be of recent formation, should be nearly of the full size of the urethra; but if the stricture has been of long duration, it should be considerably smaller. The common silver catheter is not so well adapted for the purpose as that which I now show you. You will observe that it is shorter and less curved than usual; and that it is fixed in a wooden handle, which renders the instrument more manageable than it would be otherwise. If you use an elastic gum catheter, the iron stilet should have a flattened handle, resembling that of a common sound. You should pass it as far as the obstruction, and having ascertained where it is situated, withdraw the catheter a little, a quarter of an inch for example, and then, as you pass it on again towards the bladder, keep the point sliding against the upper part of the urethra, which is towards the pubes, avoiding the lower part, which is, of course, towards the perineum. Be careful to employ no violence. If you lacerate the urethra, so as to cause hæmorrhage, you will be defeated in your object. Press the catheter firmly, but gently and steadily, against the stricture, keeping in your mind the anatomical position of the parts, and being careful to give the point of the instrument a right direction. When the pressure has been thus carefully continued for some time, the stricture will begin to relax. It will allow the point of the catheter to enter, and, at last, to pass completely through it into the bladder. In some instances this will be accomplished in the space of one or two minutes; while in others it may be necessary to persevere for a quarter of an hour. As soon as the catheter has reached the bladder, the patient's sufferings are at an end, as the bladder becomes completely emptied. If you have used the elastic gum catheter, it may be prudent to allow it to remain in the urethra and bladder for one or two days, or even for a longer period; and this will go far towards accomplishing the cure of the stricture.

If you are skilful and prudent in the management of the catheter, you will generally succeed in introducing it into the bladder; but if you fail in doing so, the attempt to introduce it may still be useful to the patient. The pressure of the catheter against the stricture, if kept up for a considerable time, exhausts the morbid irritability of this diseased portion of the urethra. The spasm becomes in a considerable degree relaxed, and if you withdraw the instrument when the patient has a violent impulse to make water, the urine will follow in a stream. Observe, that I am taking it for granted that you are careful to avoid all violence. If the membrane of the urethra be lacerated, the probability is, that the spasm will not give way; and if, under these circumstances, you persevere in the attempt to introduce the catheter, you will but aggravate the evil which it is your object to remove.

The remedy on which you are most to rely, where these mechanical means fail, is opium. From half a dram to a dram of laudanum may be given as a clyster in two or three ounces of thin starch. If this should not succeed, give opium by the mouth, and repeat the dose, if necessary, every hour until the patient can make water. According to my experience, the cases in which the stricture does not become relaxed under the use of opium, if administered freely, are very rare. The first effect of the opium is to diminish the distress which the patient experiences from the distention of the bladder. Then the impulse to make water becomes less urgent; the paroxysms of straining are less severe and less frequent; and after the patient has been in this state of comparative ease for a short time, he begins to void his urine, at first in small, but afterwards in larger quantities.

It is customary in these cases to employ the warm bath. It is, indeed, sometimes useful, but you can place no dependence on it as compared with opium. It is not sufficient that your patient should sit in a hip bath: the bath, to be at all efficient, must be complete; his whole person ought, therefore, to be immersed and he should remain in it for half an hour, or an hour, or longer, unless he previously becomes faint. Bleeding from the arm is seldom required in cases of retention of urine from stricture; but, in some instances, even where other means have failed, taking blood from the perineum by cupping gives immediate relief.

Purgatives require some time to produce their effect, and, in most cases, at the period of your being called in, the symptoms are too urgent to admit of this delay. Where, however, a stricture is chiefly spasmodic, and the retention follows the too great use of fermented liquor or spirits, I would advise you, if you are sent for on the commencement of the attack, to prescribe a draught of infusion of senna with the tartrate of potass and tincture of jalap. As soon as this has fully operated, and the bowels are emptied, give thirty or forty drops of tincture of opium by the mouth, or order an opiate clyster to be administered, and, in all probability, the attack will subside.

After all, there is no absolute rule as to the treatment of retention of urine from stricture. One person is relieved in one way, another in another; and you will do well in each case to bear in mind the particular mode of treatment which has proved of service, in order that you may at once resort to it, if you are called a second time to the same patient, under the same circumstances. In one instance, you will be able to pass a catgut bougie, and not a catheter; in another you will be able to pass a catheter, and not a catgut bougie. One individual is relieved by opium, another by the warm bath. A gentleman of my acquaintance, who was subject to attacks of this description for a considerable time, almost always began to make water after a pint of warm water had been thrown up as a clyster. To show what various treatment is necessary, I have been in the habit of mentioning the following case. A gentleman, who had been long in hot climates, laboured under an old stricture of the urethra. He was able to pass a bougie for himself; and he did this at regular periods, and for a long time experienced little or no inconvenience from his disorder. One night, however, he was seized with retention of urine, and called me out of my bed in consequence. I introduced a gum catheter, which entered the bladder with perfect ease, and drew off the urine. He called me up another night, and another, and another still; and one night he called me up twice. At last, it occurred to me that he always sent for me on the alternate nights; and on inquiry, I found that the attack of retention regularly came on about twelve o'clock, and even though the catheter had entered the bladder, the spasm did not relax, so as to enable him to make water by his own efforts, until five or six in the morning. I determined then to treat the case as we do many other intermitting and periodical diseases; and I prescribed him the sulphate of quinine. The first night after he began to take it he had an attack of retention; but he had no attack afterwards." 41.

When an operation becomes indispensable, which Sir Benjamin thinks it must *rarely* be, he is of opinion that puncturing the bladder from the rectum is applicable to the greatest number of cases. But those who operate frequently must often operate unnecessarily.

*Treatment of Stricture in the Anterior part of the Urethra.*—Sir Benjamin observes that a *stricture at the orifice of the urethra* may be dilated by means of a common bougie, or a short metallic instrument; the size of the bougie being gradually increased, and the introduction being repeated daily or on the alternate days according to circumstances. The process of dilatation is however, in many instances, attended with much inconvenience to the patient. In those cases, especially, in which the contraction began in early life, every introduction of the bougie causes considerable pain; at the same time that the disposition to contract is so great that the operation requires to be repeated almost daily. The consequence is, that the part is kept in a constant state of inflammation, and, between the disease and the remedy, is a source of incessant annoyance to the patient. In a case of this sort, which was extremely troublesome, Sir Benjamin determined at once to divide the contracted part of the urethra. This was easily accomplished by means of a pair of knife-edged scissars, one blade with a blunt point being introduced into the urethra, and the division being made in the situation of the frænum. No hæmorrhage followed the operation. A piece of lint was kept between the cut surfaces to prevent their re-union, and in about ten days they were cicatrized, being covered by what had already assumed a good deal of the appearance of a mucous membrane.

Strictures in the anterior part of the urethra, but behind the orifice, require to be mechanically dilated, by the introduction of bougies or metallic instruments. Sometimes the patient obtains relief on very easy terms, the dilatation being readily accomplished, and the use of a bougie once in three or four days being sufficient to prevent a recurrence of the contraction. At other times, however, the disposition to contract is so great, that it becomes necessary to introduce the bougie once or twice daily; and indeed, Sir Benjamin has known cases in which the patient was seldom able to expel his urine until the bougie had been employed.

After speaking of the *common wax bougie*, which Sir B. Brodie thinks the preferable instrument where it can be used, he observes that he does not employ the common flexible metallic bougies, as they are liable to lose the shape which you have given them during their introduction, and, in fact, are at the same time too flexible and too inflexible for any useful purpose. Sir Benjamin's bougies, if of a small or middle size, are made of solid silver; the larger ones of silver or steel, or steel plated, or of a composition similar to, but firmer than, that of the flexible metallic bougie. These sounds should be very slightly curved, and for ordinary cases not more than eight inches and a half or nine inches long, exclusive of the handle. You may use them as you would use the common bougie for the purpose of gradually dilating the stricture, beginning with one of a small size, and gradually proceeding to those which are larger. These metallic instruments are applicable: 1st, to cases of old and indurated strictures, which the common bougie is incapable of dilating; 2dly, to those in which, in consequence of some improper management, a false passage has been formed, into which the point of a common bougie will easily penetrate, but which an inflexible instrument may be made to avoid; 3rdly, to those in which, from long-continued disease, and without any previous mismanagement, the urethra has become distorted, and its surface irregular; and 4thly, to several recent cases in which the smooth polished metallic surface gives less pain to the urethra, and is less likely to induce spasm, than the softer but less smooth surface of a common bougie.

In very old and inveterate cases, where other means have failed, a full-sized instrument will often succeed. "The sound should be rather above than below the middle size. Of course the same rule in this respect does not apply in every instance, but that which I generally find it most convenient to employ has only a moderate curvature. It is made of silver, fixed in a flat wooden handle, being nine inches in length from the handle to the point; no part of it is more than one fifth part of an inch in diameter, and at the point the diameter is reduced to one sixth of an inch.

In using the sound you should pass it carefully as far as the stricture, and then press the point firmly and steadily against it, taking care that it is directed in the line of the urethra towards the bladder. The pressure is to be continued for five, ten, or fifteen minutes, or even longer, according to circumstances; and this process is to be repeated once in two or three days. If a false passage exists, it is probably on the lower part of the urethra towards the perineum; and it is in this situation that, by careless management, one may be easily made. To avoid this mischief, you must direct the point of the sound especially to the upper part of the stricture next the pubes. The pressure should be as much as can be made without the urethra being lacerated, and without inducing any considerable degree of pain. In some instances the stricture has little or no sensibility, in others it is exquisitely tender; and in the latter cases the pressure should be very trifling at first, but it may be gradually increased as the tenderness subsides (as it will do) under its influence.

The result of this treatment is, that at each operation the anterior part of the stricture seems to become relaxed to a greater or less extent; and that at last the instrument penetrates entirely through it and enters the bladder. The period at which this happens, of course, varies in different cases. The permanent change of structure may be trifling, the stricture being chiefly spasmodic, and one or two applications of the sound may be sufficient. There may be much gristly induration, occupying a considerable portion of the urethra, and many applications may be required. A patient was under my care, in whom the stricture was surrounded by a mass of hard substance, which could be distinctly felt in the perineum, apparently an inch or an inch and a half in length. The stream of urine was of the smallest size, and varied so little that it was evident that there was little or no liability to spasms. For many years before I was consulted no instrument had been made to enter the bladder; and the ordinary methods, after a long trial, failed in my hands, as they had done in those of others. At last I succeeded by the method which I have just described, but not until I had persevered in it for many months."

After describing the method of treating stricture by means of the *gum catheter*, which is left in the urethra, Sir Benjamin points out the cases to which he believes it to be applicable. They are, 1st. Where time is of much value, and it is of great consequence to the patient to obtain a cure as soon as possible.

2dly, Where a stricture is gristly and cartilaginous, and therefore not readily dilated by ordinary methods.

3dly, Where, from the long continuance of the disease, the urethra has become irregular in shape, or where a false passage has been made by previous mismanagement. Under these circumstances, if you can succeed in introducing a gum catheter, and let it remain for a few days in the bladder, you will find your difficulties at an end; the irregularities will disappear, and the false passages will heal.

4thly, Where a severe rigor follows each introduction of the bougie. This disposition to rigor is such, that it is sometimes impossible to proceed with the treatment in the ordinary way. Observe, in these cases, when the rigour takes place. It seldom follows the use of the bougie immediately. It almost always occurs soon after the patient has voided his urine, and seems to arise, not as the immediate effect of the operation, but in consequence of the urine flowing through the part which the bougie has dilated. Now, if, instead of a bougie, you use a gum catheter, and allow it to remain, the urine flowing through the catheter, the contact of it with the urethra is prevented, and the rigor is prevented also.

Sir Benjamin conceives that the *caustic bougie* is applicable to the following cases: 1st, Those of spasmodic stricture, where two or three applications of the caustic may be sufficient to relieve all the urgent symptoms. 2dly, Some cases of old stricture, in which there still is a considerable disposition to spasm. In these last cases apply the caustic two or three times, and no oftener. It will probably relieve the contraction as far as it is spasmodic, and thus enable you to proceed more advantageously, with the use of the bougie or metallic sound. 3dly, The caustic may be used very properly in some cases of stricture which are endowed with peculiar irritability, in which every application of the common bougie induces severe pain, or brings on spasm, preventing it entering the stricture. Two or three applications of the caustic may be sufficient to deprive the stricture of that unnatural sensibility which otherwise would have foiled your efforts to effect a cure. Yet our author seldom employs caustic for these reasons: 1st, Although the caustic often relieves spasm, it also very often induces it. It is true, that in many instances it enables a patient to make water with more facility; but in many instances, also, it brings on a retention of urine. 2dly, Hæmorrhage is a more frequent consequence of the use of the caustic than of the common bougie, and it sometimes takes place to a very great and to an almost dangerous extent. 3dly, Where there is a disposition to rigors, the application of the caustic is almost certain to produce them; and frequently the application of the caustic induces rigors where there had been no manifest disposition to them previously. 4thly, Unless used with caution, the application of caustic may induce inflammation of the parts situated behind the stricture, terminating in the formation of abscess.

*Division of Stricture.*—Sir Benjamin remarks of Mr. Stafford's method of dividing strictures that there are very few cases for which it can be needed. But he relates an instance in which he adopted a modification of it with success.

*Case.*—"A man, forty years of age, was admitted into St. George's Hospital, in the year 1835, labouring under a stricture, near the bulb of the urethra, complicated with a fistulous opening in the perineum. When he voided his urine, a very small quantity came away by the urethra, the greater part being discharged by the perineum. The disease had existed for more than twenty years, and the abscess in which the fistula had originated had followed an injury received while riding on horseback thirteen years ago. For many years no instrument had been passed through the stricture. At last he became a patient under the late Mr. Earle, in St. Bartholomew's Hospital, where he remained under treatment for five months, but with no more success than formerly.

Finding after repeated trials that no instrument could be made to penetrate through the stricture, with the concurrence of my colleagues, I performed the following operation:—

The patient having been placed in the same position as in lithotomy, a full-sized plaster bougie was introduced, and held by an assistant with its extremity resting against the stricture. I then made an incision in the perineum, dilating the fistulous sinus, and laying open the membranous part of the urethra as far forward as the stricture, the exact situation of which was marked by the bougie. The bougie was then withdrawn, and an instrument was introduced in its place, consisting of a straight silver tube, closed at its extremity, except a narrow slit, through which a small lancet could be made to project by pressing on a stilet which projected the handle of the instrument. The round extremity of the tube being pressed against the anterior part of the stricture, I applied the fore-finger of the left hand, introduced through the wound in the perineum and urethra, to its posterior surface. The pressure of the instrument being distinctly communicated to the finger through the substance of the stricture, the lancet was protruded, and the stricture was divided. A silver catheter was then easily introduced through the urethra and the divided stricture into the bladder, and allowed to remain there. The urine of course flowed through the catheter. At the end of two days the silver catheter was removed, and replaced by one of elastic gum. The wound in the perineum gradually healed, and the patient ultimately recovered, making water in a full stream, and being able to introduce a sound of a full size into the bladder, so as to prevent a recurrence of the contraction.

The instrument used on this occasion was ten inches in length, exclusive of the handle, and rather more than one quarter of an inch in diameter. The lancet measured three sixteenths of an inch at its broadest part; it terminated in a sharp point, and could be made to project by pressing a button on the other end of the stilet to which it was attached to the length of half an inch, returning to its place within the silver tube when the pressure was withdrawn by the action of a spiral spring. In using it, one cutting edge of the lancet was directed towards the pubes, the other towards the perineum. The advantages of dividing the stricture by this method, as compared with other methods of operating, are 1st, that the free opening made in the perineum prevents all danger from infiltration of urine; 2dly, that the fore-finger of one hand, being applied to the posterior surface of the stricture, serves as a guide for the lancet, and enables you, with the exercise of a little skill and caution, to make an exact division of the stricture." 67.

*Fistulæ in Perineo.*—After pointing out the necessity for freely dilating the natural passage, before the fistula can have a chance of closing, Sir Benjamin adds his latest experience. "I formerly," he says, "have advised the patient never to void his urine without the aid of the catheter; but I am now inclined to believe that the irritation thus kept up tends, on the whole, to delay rather than to expedite the cure. At other times I have kept the patient in bed for some weeks, with an elastic gum catheter constantly in the urethra and bladder; but I cannot say that, with my present experience, I have much more faith in this mode of treatment than in that which I mentioned before. After a few days the urine generally begins to flow by the side of the catheter, which does not therefore answer the purpose for which it was introduced, of preventing its escape by the sinus. Then in many cases the catheter causes an abundant suppuration of the urethra; and the purulent discharge, finding its way into the sinus, prevents it from closing as much as it would be prevented by the contact of the urine."

*Blind Fistula in Perineo.*—Sir Benjamin advises the following method of treatment. Watch for the opportunity when matter is collected in it, and then establish an external opening by dividing the integuments over it with a lancet, so as to convert it into a fistula of the ordinary kind. There are some of these cases, however, the treatment of which requires a more particular explanation. A patient may apply to you who perhaps has had gonorrhœa formerly, followed by a slight obstruction of the urethra, complaining at the same time of a discharge from the urethra, which he calls an obstinate gleet. You examine the perineum, and you find in it a small tumor, not larger than a horse-bean or filbert. It is at some distance from the surface, and the patient says that it has been co-existent with the gleet, and that it is sometimes inflamed and tender. Now this little tumor indicates the existence of a blind fistula. There is a small orifice in the urethra, and a narrow channel leading from it into the centre of the tumor; and every time that the urine flows, a very small quantity finds its way into this channel, escaping from it immediately afterwards by regurgitation into the urethra. In consequence of the smallness of the cavity, and the quantity of solid matter deposited on its outside, the fluctuation of fluid in it is not perceptible. I have known this state of things to continue, producing more or less occasional inconvenience, for many years. The first thing necessary to the cure is to make an opening in the perineum leading into the cavity in the centre of the tumor. But this may not be very easily accomplished, on account of the smallness of the cavity. You should introduce the lancet somewhat obliquely, so as to divide the tumor as nearly as possible through its centre. Then introduce some lint, so as to prevent the wound uniting by the first intention. After three or four days you may remove the lint, and then you will ascertain whether you have done what was required, by observing whether, when the patient voids his urine, any portion of it flows through the opening which you have made. If this be the case, nothing further is required than that the stricture should be dilated in the usual way. If, however, no urine flows through the opening, you may proceed thus:—Introduce a piece of caustic potash through the wound into the centre of the tumor, so as to make a considerable slough. A portion of the tumor being thus destroyed, the probability is that, when the slough has separated, it will be found that the central cavity is exposed, and that you have accomplished the object which you had in view.

*Obstructions of the Urethra, arising from Mechanical Injury.*—Sir Benjamin makes some valuable observations on this subject.

These obstructions may occur in various parts of the urethra, and may be produced in various ways.

1. A boy contrived to slip his penis into a small metallic ring. The swelling of the glans made its removal difficult, and, when this was at last accomplished, it had caused ulceration of the skin and *corpus spongiosum*, extending into the urethra. As the ulcer healed, the urethra became contracted; and when the patient was admitted into the hospital some time afterwards, there was a small fistulous orifice in the middle of a hard cicatrix, through which the greater part of the urine was discharged, while a common probe was with difficulty passed from the external orifice through that portion of the urethra which was included in the cicatrix.

2. A more frequent seat of the obstruction is that part of the urethra which is immediately below the pubes, where the mucous membrane is especially liable to suffer from a blow, compressing it against the hard substance of the bone. In some cases these obstructions are formed where there is no evident injury of the integuments or the other superficial parts of the perineum.

3. In other cases, a deep wound of the perineum may extend into the urethra. If the urethra be only partially divided, Sir Benjamin concludes, that no more mischief will ensue there after the operation of lithotomy; but if the division be complete, it is difficult to conceive that in the progress of the cicatrization a contraction of the urethra shall not ensue.

4. But there are cases of more frequent occurrence, in which a blow on the perineum has lacerated the urethra, contused the parts between it and the skin, caused an effusion of blood into the perineum and scrotum, some portion of urine becoming infiltrated into the cellular membrane afterwards; the result of the whole being the formation of an abscess, and the destruction of the injured parts by sloughing to a greater or less extent. Here, as the sore heals, a hard gristly cicatrix is generated, adhering to the pubes, with an orifice in the centre, through which the whole or the greater part of the urine is discharged.

The condition of a patient under the circumstances which have been described is much worse than that of one who labours under a perineal fistula connected with an ordinary stricture of the urethra. The difficulty of voiding the urine is more constant; it is liable to be increased, so as to become a complete retention, from attacks not of spasm, but of inflammation, producing at the time much pain in the perineum, and followed by a fresh accumulation of matter beneath the cicatrix; and, in addition to all this, the treatment of these cases is not less troublesome to the surgeon than it is distressing to the patient, and for the most part does not lead to the same satisfactory results as that of ordinary stricture.

Sir Benjamin gives the following directions with regard to *Treatment*.

" In all cases," he says, " in which there is reason to believe that the urethra has been divided or lacerated in consequence of an injury inflicted on the perineum, it is the duty of the surgeon not only to look at the great and immediate danger, but to guard against future ill consequences; and much may be done at this period towards preventing a most serious inconvenience, which would be relieved with difficulty afterwards. If there be a penetrating wound, in which the urethra is probably implicated, an elastic gum catheter should be introduced with the least possible delay, and allowed to remain in the urethra and bladder until the healing of the wound is far advanced, or, at all events, until it is ascertained that the urethra has not suffered; the catheter being, however, occasionally removed for a limited time, if it seems to act as a source of irritation.

In cases of contusion of the perineum, when the effusion of blood in the perineum and scrotum, and more especially the discharge of blood from the urethra, or any other circumstances, lead to the suspicion that the urethra has been lacerated, the same treatment should be had recourse to: the gum catheter should be introduced as soon as possible, and allowed to remain for at least some days after the occurrence of the accident. The extravasation of blood does not in itself justify the making an incision in the perineum; and indeed, according to my experience, there can be no worse practice than that of making an incision in a case of simple ecchymosis, either in this or in any other situation. But where such extravasation exists, there is always reason to apprehend that there may be further mischief; the progress of the case, therefore, should be carefully watched, and on the first appearance of any symptoms which might be supposed to indicate that urine had escaped into the cellular membrane, or that suppuration had begun to take place, a staff should be introduced into the urethra instead of the gum catheter, and a free incision should be made from the perineum into it, the gum catheter being replaced afterwards.

But it may be that these measures of precaution have not been adopted in the first instance, and that you are not consulted until after the lapse of a considerable time, when the wound or laceration of the urethra is already healed, leaving the urethra contracted in the situation of the cicatrix. Here you may perhaps succeed in gradually dilating the urethra, as where there is an ordinary stricture. But in a case which I have already mentioned, I have stated that 'this was not accomplished without a great deal of local and constitutional disturbance;' and so it has been in all the cases of this kind which have fallen under my observation. Nor will the occurrence of such difficulties be a matter of surprise to any one who bears in mind that here the object is to dilate, not a genuine stricture, but a cicatrix, of the urethra, and who has observed how the cicatrix of an old sore leg inflames and cracks when the subjacent muscles begin to increase in bulk from exercise, or how the endeavour to extend forcibly the contraction after an extensive burn produces the same result. It may be that these difficulties are insuperable under the method of treatment by simple dilatation; and under these circumstances, a small staff having been introduced into the bladder, the cicatrix of the urethra should be divided by an incision from the perineum, a gum catheter being introduced afterwards, and allowed to remain until the wound is healed over it. But even then much remains to be accomplished. The cicatrix has still a greater disposition to contract than an ordinary stricture; the bougie or catheter must be had recourse to almost daily, and the patient must be contented if he can persevere in the use of instruments of a moderate diameter, as the urethra will invariably resent the attempt to keep it dilated by those of large dimensions." 83.

The condition of the patient is improveable, where the injury of the urethra is limited. But where there has been actual loss of some portion of the canal, the patient must either be content to void the whole of his urine by the perineum, or submit to an operation for establishing a communication between the anterior and posterior portions of the urethra. This operation is best explained by a case.

"A young man, in making a leap on horseback, received a violent blow on the perineum from the pummel of the saddle. The immediate consequence of the injury was hæmorrhage from the urethra, and this was followed by extravasation of urine and sloughing of the perineum to a considerable extent. A catheter was at first introduced into the bladder, but it was afterwards removed. The sloughs having separated, the sore in the perineum gradually closed, a small fistulous opening only being left immediately behind the scrotum, through which the whole of the urine was discharged. He was in this state seven months after the occurrence of the accident, when he arrived in London, and Mr. Baker advised him to have my opinion on his case.

On introducing an instrument into the urethra I found an obstruction of the canal immediately below the pubes. Several ineffectual attempts having been made to penetrate the obstruction in the usual manner by bougies and sounds of various sizes, I had recourse to the following operation:—The patient having been placed in the same position as in lithotomy, a staff was introduced into the urethra, and held by Mr. Hilles, who, with Mr. Baker, assisted me in the operation, with the extremity of it resting against the obstruction. I then made an incision in the perineum, extending backwards from the part in which the staff was to be felt, in the direction of the prostate gland. It was now evident that not less than three-quarters of an inch of the urethra was deficient below the pubes; the place of it being occupied by a rigid cicatrix. This having been divided longitudinally by the point of the scalpel, I was enabled, though not without some difficulty, to pass the staff from the part at which the extremity of it rested, into the sound portion of the urethra towards the bladder, and then into the bladder itself. The staff was then withdrawn, and an elastic gum catheter having been substituted for it, the latter was allowed to remain in the urethra and bladder. On the ninth day after the operation, there being some degree of irritation at the neck of the bladder, the catheter was removed, being reintroduced, however, after two days more. From this time it was removed at intervals, which were sometimes longer, sometimes shorter, according to circumstances. The wound in the perineum gradually healed, and in less than ten weeks from the time of the operation was reduced to the diameter of a small pea. The patient was now able to introduce a silver catheter of the size of his urethra into the bladder without difficulty, and he repeated this operation so as to draw off his urine three or four times daily. When he voided his urine without the catheter, by placing the point of his finger on the opening in the perineum, he was enabled to discharge the whole in a sufficient stream by the urethra."* 86.

While treating of chronic inflammation of the bladder, and speaking highly of the *decoction* of pareira brava, Sir Benjamin observes :—The infusion of *pareira brava,* which has been introduced into the last Pharmacopœia of the College of Physicians, does not at all answer the purpose of the decoction, and is nearly useless. The decoction is made thus :—Take half an ounce of the root of the *pareira brava,* add three pints of water, let it simmer gently, near the fire, until reduced to one pint. The patient is to drink from eight to twelve ounces of this decoction daily. If so large a quantity of liquid should be offensive to the patient's stomach, he may take the extract of *pareira brava* instead, twenty-five or thirty grains being equal to half a pint of the decoction.

*Incontinence of Urine.*—We find some remarks on this head which we may notice. After stating that incontinence may be the result of wound or sloughing of the neck of the bladder, or (which is most common) of an over-distended bladder, from stricture, or enlarged prostate, or paralysis, Sir Benjamin goes on to say :—" there are some cases of paralysis in which there is incontinence of urine although the bladder is empty, as if the same cause which rendered the lower limbs paralytic rendered the bladder incapable of distention. For example, a gentleman, sixty-three years of age, swallowed by mistake a bottle of liniment, of which the

---

* " The last report which I had of this patient was six months after the operation, and to this effect: ' that he had continued to improve, and expected in the course of a fortnight to be as well as ever.'

Since the manuscript of this Lecture was prepared for the press, a case very similar to that described above has come under my care, in the person of a young man nineteen years of age. He had received an injury of the perineum in leaping over a gate about a year ago. Three quarters of an inch of the urethra below the pubes seemed to be deficient. I made an artificial canal, joining the anterior and posterior portions of the urethra to each other, by perforating the cicatrix with the instrument having the concealed lancet, described at page 67, leaving an elastic gum catheter in the urethra and bladder afterwards. At this time, about ten weeks after the operation, the patient voids his urine by the urethra in a full stream, without pain or difficulty, no more than a few drops escaping by the opening in the perineum. A common plaster bougie may be introduced readily into the bladder. Mr. Guthrie saw this patient with me, and lent me his assistance at the operation."

tincture of cantharides was a principal ingredient. In about three quarters of an hour an emetic was administered; nevertheless he was immediately afterwards affected with paralysis of the lower extremities, and inability to void his urine. For the first fortnight he was under the necessity of having his urine drawn off at stated periods. After this he regained the power of making water, but was tormented by an incessant desire to do so. When I was consulted, four years after the commencement of the attack, he was able to walk with the assistance of crutches. At times he had a sudden and irresistible impulse to void his urine, and expelled a small quantity by a voluntary effort; but at other times it flowed involuntarily, without his being conscious of what happened, so that his clothes were as wet as possible. On introducing a catheter, I found that the bladder was empty. It may be supposed, that in this case something was to be attributed to the peculiar nature of the stimulus which had been swallowed. I have, however, observed the same thing in some cases of paralysis of the lower limbs, arising from other causes. I have occasionally seen what was called a case of incontinence of urine in young women having a disposition to hysteria: but from a close observation of such cases, I am led to believe, that the discharge of urine, although involuntary in appearance, is not involuntary in reality; and that this symptom, like many other hysterical symptoms, is to be referred to a mis-direction of the power of volition, and not to the actual want of it. The case which I am about to mention seems to confirm this view of the subject. A lady, twenty years of age, for the last ten or eleven years had been troubled with a constant discharge of urine. It flowed (as she said) without her being able to prevent it while she sat in her chair, and while she was walking; so that she was quite unfit to live in society, or even in her own family. All the plans of treatment, recommended by myself and others, proved inefficacious. At last, on account of this infirmity, it was thought advisable that she should be separated from the rest of her family, and she was sent to reside at a distance from them. After some time she was seized with an urgent desire to return home, and immediately she regained the power of retaining her urine. She continued well when I heard of her some time afterwards."

Sir Benjamin thinks the incontinence of urine of children involuntary in the first instance, but almost unconsciously voluntary ultimately. His mode of treatment does not seem very satisfactory to himself, or successful. It is as follows:—

"It is reasonable to suppose, that those children whose urine is of a too stimulating quality, in consequence of an excess of lithic acid in it, may be more liable to this kind of incontinence than others; yet I must say, that my endeavours to relieve it by the exhibition of alkalies and purgatives, combined with a regulated diet, have been generally unsuccessful. A blister applied over the *os sacrum*, and repeated according to circumstances, is a more effectual remedy. Sir Charles Bell has observed, that children are more liable to this troublesome symptom when they lie on their back than when they lie on the face or side. This may explain, in part at least, the good arising from the blister. The same object may be attained by making the child wear, during the night, a machine, so contrived as to prevent him lying in the supine posture. I do not know that you can absolutely rely on this method for the patient's cure, but it may often be employed advantageously, in combination with other methods of treatment. In some cases, the discharge of urine is periodical, returning at the same hour of the night and morning. You may then direct the nurse to take the child out of bed, so as to give him the opportunity of making water about an hour before; or if the patient be older, he may be provided with a clock, having a loud alarum, for the purpose of awakening him from his sleep at the proper moment. Under the same circumstances the sulphate of quinine may be administered with great advantage. But in no instance are any of these remedies likely to be successful, unless the patient himself feels a strong desire to be relieved; and unfortunately this desire is too often wanting, long habit gradually reconciling the mind to this, as it does to many other inconveniences, until, at last, it seems to be a matter of indifference whether relief is obtained or not. I have heard of young persons being cured of this kind of incontinence of urine by applying caustic to the neck of the bladder, and by the introduction of bougies or catheters. If these methods of treatment produce any effect, I suspect that it is simply by annoying the patient, and by giving him that strong desire to be relieved, which I have just mentioned as the first step towards recovery." 118.

After giving an account of inflammation of the prostate gland, Sir Benjamin observes, that he has seen a case in which the symptoms yielded to iodide of potassium. The case in question was as follows :—A gentleman, aged 31, complained of pains in the perineum, hypogastrium, and back of the pelvis, extending down the thighs. "He had a sense of obstruction in the rectum on the passage of the fæces. He was tormented by the desire to void his urine more frequently than is usual; but he had no difficulty in voiding it: he could empty his bladder by his own efforts, and the urine was transparent and healthy. The urethra was free from disease; but the prostate gland, when examined from the rectum, was found to be enlarged to two or three times its ordinary size." He traced the disease to a severe gonorrhœa ten years before—the disease had been severe for three or four years. Sir Benjamin prescribed two grains of the iodide of potassium thrice daily. This seemed to remove the complaint.

Our straitened limits will not suffer us to pursue the other subjects treated of in this able work seriatim. We must jump to the concluding lecture on LITHOTRITY.

Sir Benjamin gives a slight sketch of the modern history of lithotrity, which establishes the fact that no man can be said to have wholly invented *and* applied the present lithotritic apparatus. On the contrary, like most other valuable discoveries it has been perfected only by successive and gradual steps.

Sir Benjamin alludes to Baron Heurteloup's application of the hammer. He doubts its superiority to the screw, and believes that "there is nothing that can be done by the hammer which may not be done quite as effectually by the screw, while the latter method is not liable to many serious objections which may be urged against the former."

Sir Benjamin gives a sketch of the instruments which he employs. It is Weiss's screw lithontriptor—its average length about eleven inches, exclusive of the handle. But he has one instrument, made for a particular occasion, thirteen inches long. It is well to have several instruments, of various sizes and shapes.

"For calculi of a small size the construction (except as to the addition of the screw) need scarcely differ from that of the common urethra-forceps which I described formerly; but for larger ones the opposite blades of the forceps should be furnished with projections or teeth; and for those of a still larger size you will find it convenient to be provided with a forceps, in the fixed blade of which there is a longitudinal slit, while there is a corresponding wedge-like projection, fitted to enter the slit, in the opposite surface of the movable blade." 359.

Through the slit, fragments drop into the bladder, so that a large stone may be crushed. The diameter of the instrument must vary with that of the calculus and of the urethra. It should, usually, be as large as the canal will admit. It should be cylindrical, except in the handle. But it is well to be provided with one, the blades of which beyond the curvature are somewhat flattened, and in proportion broader than elsewhere. It is useful for seizing and crushing the smaller fragments.

So much for the instruments. Sir Benjamin next speaks of the measures preparatory for an operation.

The forceps should never be used in a bladder which will not retain at least six ounces of water. If the organ be irritable, the patient must be placed in the recumbent posture and the catheter introduced every, or every other day, with the view of injecting some ounces of tepid water. In this way the bladder will gradually become educated to containing liquid. If chronic inflammation exists with a deposit of adhesive mucus, the remedies for that state must be adopted. An abundant formation of this mucus forms a great objection to the operation—because it indicates an unfavourable condition of the bladder, and because the fragments are apt to be entangled in it. But where there is only little mucus, this may altogether disappear after the first crushing of the calculus.

The urethra should be dilated so as to admit an instrument of sufficient size and strength. If contraction within or behind the glans cannot be easily stretched, it must be divided with a bistoury.

There is a tumid condition of the prostate, impeding the introduction of the instrument, and rendering the part very liable to bleed. It subsides after a few days of constant repose in the recumbent posture. Sir Benjamin has "observed this state of things to exist especially after travelling in a carriage; and it forms one of many reasons, where the patient has come from a distance, for not recommending the operation to be had recourse to until he has had ample time to recover from the fatigues of his journey."

Let us pass to the operation.

The patient should be on his back on a sofa, or on the edge of the bed, with a thick cushion under the pelvis, to elevate the neck of the bladder. A silver catheter is then introduced, and as much tepid water as can easily be borne is injected.

"The catheter used for this purpose should be provided with a stopcock, and the extremity of it should not be prolonged a great deal beyond the curvature. It may then be used, not only as a catheter, but also as a sound, for the purpose of exploring the bladder, and ascertaining in what part of the bladder the calculus is, at that time, lodged. This knowledge is always useful, but it is by no means indispensable; and I have often been able to seize a small stone with the forceps which I had not been able to detect by other methods previously. The injection of the bladder having been completed the catheter is to be withdrawn, and the lithotrity-forceps is to be introduced in its place. In consequence of the peculiar shape of the latter this is less easily accomplished than the introduction of the catheter. The mere depression of the handle is not always sufficient to make it enter the bladder; and it is often necessary at the same time to apply a moderate but steady force during the time that the curved part of the instrument is passing through the neck of the bladder. This is especially the case where the prostate gland is in any degree enlarged. You will know when the instrument has fairly entered the bladder by the facility with which you can move it in any direction, and by your being able to open the bladder to any extent without giving the patient pain. You may then explore the bladder with the forceps, and endeavour to ascertain the exact situation of the calculus in it. If it be lying on one side, by opening the blades, and then gently and cautiously turning them towards it, you will probably be enabled to seize it. If you do not succeed by this method, by the following you will rarely fail.

Raise the handle of the forceps so as to bring the convexity of the fixed blade in contact with the posterior part of the bladder; then open the movable blade, at the same time making a moderate pressure downwards in such a manner as to depress the bladder towards the rectum. The instrument being then gently shaken by a lateral motion of the hand, the calculus, in whatever part of the bladder it may be situated, will roll between the blades and will be seized by closing them. Having been thus carefully secured, by turning the screw it is broken into fragments. The whole of this is a very simple process, requiring but little practice to make you a perfect master of it. When the calculus has been once broken, the fragments are to be seized and crushed in the same manner. They will fall one after another into the grasp of the forceps; and there is no limit to the number that may be crushed at one time, except what is afforded by the diameter of the urethra. Every fragment that is crushed adds to the accumulation of calculous matter; and if the accumulation be very large, it becomes difficult, or impossible, to withdraw the instrument without injury to the membrane of that canal. The marks on the handle of the instrument inform you of the exact extent to which the blades are separated; and you must use your own discretion, founded on your knowledge of the size of the urethra, as to the point at which you should stop. The forceps first used being then withdrawn, you may use a second, and even a third, in the same manner; and thus you may not only crush a great number of fragments at one operation, but you may remove from the bladder a great deal of what has been crushed." 365.

The forceps should be withdrawn very slowly and gently. When as much as is prudent has been done, the catheter should be re-introduced, and the bladder emptied.

"Another syringe-full of water may then be injected, which the patient may be left to void by his own efforts, or which may be drawn off by means of a large catheter, with two apertures near the extremity of sufficient size to allow some of the smaller fragments to escape through them." 366.

Sir Benjamin thinks it very unsafe to allow a patient to walk after the operation. He should remain on his bed or sofa. Whenever there has been rough usage of the urethra, at all events, an opiate should be given after the operation, and the bowels should be attended to.

The patient should be watched lest a fragment stick in the urethra, and occasion retention. But this is unlikely if the patient reposes, and the fragments seldom find their way into the urethra for the first day or two. After this they begin to pass away with the urine, and the patient should collect them.

If in the operation the stone is of large size, the forceps with the slit in one blade and wedge in the other, should be made use of. Scarcely any calculus will resist this.

A small calculus may be got rid of by a single operation. A larger one requires several. It should not be repeated until the patient has recovered from the effects of the preceding one, nor should it be delayed long afterwards.

Of course, it is of the first importance to get rid of every fragment. The bladder should be explored with sound *and* forceps, at least twice after the cure seems complete. With this precaution, when the patient can empty the bladder by his own efforts, the chance of a fragment remaining is very slight.

"But it is quite otherwise in those cases in which the patient, in consequence of an enlargement of the prostate gland, is unable to empty the bladder by his own efforts. Hence only a small portion of the crushed calculus will come away in the stream of urine and you must be satisfied with washing out the remainder of it through the catheter by repeated injections of tepid water. Mr. Weiss has invented a forceps which, when the blades are opened in the bladder, answers at the same time the purpose of a catheter, and this is often very useful; still on ordinary occasions you will find nothing to answer the purpose better than a silver catheter of as large a size as the urethra, with two very large apertures near the closed extremity, not placed laterally, as in ordinary catheters, but one on the anterior or concave, and the other on the posterior or convex surface. It may indeed be said, that, in the cases now referred to, this kind of operation ought not to be recommended. But it will sometimes happen, that although the patient may have had no difficulty of emptying the bladder before the operation, the prostate may be rendered tumid in consequence of its being irritated by the repeated introduction of instruments, so that he is unable to empty the bladder afterwards. Besides, although this state of things adds to the difficulty of the operation, it is not in itself sufficient to prevent it being brought to a successful termination; and in cases in which there is good reason to believe that the calculus is of a small size, it forms no objection to it." 369.

Sir Benjamin next adverts to the inconveniences or dangers of lithotrity.

1. Hæmorrhage. This may arise from the forcible passage of the lithotrity forceps through the neck of the bladder. Sir Benjamin has known it discolour the urine for two or three days. But it has never interfered with the operation.

2. Rigors may follow lithotrity. The rigor is usually produced by the stretching of the urethra by the withdrawal of the forceps; or it may be occasioned by a fragment of calculus sticking in the urethra. A dose of opium may prevent the rigor altogether, or defer it till next day. Rigors do not appear to interfere materially with the patient's recovery.

3. Sir Benjamin refers to two cases in which fragments of calculus impacted in the urethra gave rise to urinous abscess in the perineum. One patient died in two months with symptoms of diseased kidney. The other patient got well.

4. Sometimes the patient suffers from pain in the whole canal of the urethra, from the simultaneous escape of many fragments. Sometimes he labours under great irritation of the bladder, apparently from a fragment lodged near its neck. He may have complete retention, but Sir Benjamin has never seen it last for any time.

The patient should partake plentifully of diluting drinks. If fragments lodge, a middle-sized catheter may be introduced into the bladder, when they may either be dislodged and come away, or be pushed back and afterwards crushed. Sir Benjamin has removed fragments from the anterior part of the urethra by long slender forceps. It *might* be necessary to make an incision in the penis or the perineum. Sir Benjamin, however, believes that if perfect repose is enjoined after the operation, the passage of fragments will seldom occasion serious inconvenience.

5. Inflammation of the mucous membrane of the bladder may occur. It generally subsides spontaneously in two or three days, or continues till a fragment is either discharged from the urethra or pushed back into the bladder by the catheter. In one instance Sir Benjamin saw this inflammation prove fatal. The stone had been large—perhaps the patient had not been kept quiet.

On the whole Sir Benjamin is of opinion that lithotrity has great advantages over lithotomy. He touches on the cases to which it is not applicable.

1. In boys, under the age of puberty, lithotomy is too successful to be abandoned. The urethra is not wide enough to be favourable for lithotrity.

2. Lithotomy is attended with little danger in the female, while her short and wide urethra admits too readily of the escape of the injected water by the side of the lithotrity forceps.

3. Large stones are not well adapted for lithotomy, but they are still worse adapted for lithotrity. Sir Benjamin inquires whether it would not be well to crush first and cut afterwards.

4. Lithotrity is not well adapted for cases of enlargement of the prostate gland, where the patient cannot empty the bladder, unless the calculus is small; *then* the fragments may be washed out of the bladder through a large catheter. When the tumor of the prostate projects into the bladder, it is difficult to elevate the handle of the instrument sufficiently to seize the stone readily.

5. Lithotomy is very fatal where the kidney is diseased, principally, Sir B. Brodie supposes, from the loss of blood that it entails. Crushing he thinks *safer*. But any shock to the system must be hazardous, and it must, usually, be more advisable to palliate.

After a case, Sir Benjamin sums up what he has to say in favour of lithotrity in these words:—

"With the exception of such cases as those which have been enumerated, there are few to which this method of treatment may not be advantageously applied. It may be said that the exceptions are numerous; but they are the result chiefly of delay. If a patient seeks the assistance of a competent surgeon within six or even twelve months after a calculus has descended from the kidney into the bladder, the urine having remained acid, it will rarely happen that he may not obtain a cure by a single operation, and with so small an amount of danger that it need scarcely enter into his calculations. As time advances, the facility with which he can be relieved diminishes, and after the lapse of two or three years, especially if the urine has become alkaline, it is probable that the calculus will have attained such a size as to render the old operation preferable, and that the access of disease in the bladder or kidneys may render any operation hazardous. It would be absurd to say, and it would be unreasonable of human-kind to expect, that an operation which has for its object to relieve them of a disease so terrible as that of a stone in the bladder, can be always free from inconvenience, and difficulty and danger. Nevertheless, from what experience I have had, I am satisfied that the operation of lithotrity, if had recourse to only in proper cases, is not only much more successful than that of lithotomy, but that it is liable to fewer objections than almost any other of the principal operations of surgery." 379.

It must be owned that this is a favourable picture of lithotrity. The lights that characterise it contrast strongly with the sombre colours in which the operation has been drawn by Velpeau. To what is this due? May it not be owing to the careful selection of appropriate cases by the English surgeon—to the judicious management of patients during and after the operation? The candour of Sir Benjamin Brodie is sufficient guarantee for the fidelity of his statements—his judgment and experience, for the soundness of his inferences. If he speaks so well of lithotrity, it must be a valuable addition to surgery.

We cannot dismiss these Lectures without again recommending them to our readers. They bear the impress of an exact and philosophic mind, and are a model of that sober and healthy observation so eminently characteristic of a practical man. We find in them none of the extravagant views, the splitting of hairs and ridiculous refinements, the overdone erudition or the superficial flippancy which constitute the faults of our present medical literature. And last, not least, the doctrines are those of a *British surgeon*, stamped with the national features of straight-forwardness and common sense.

